# Ectopic Lymphoid Follicle Formation and Human Seasonal Influenza Vaccination Responses Recapitulated in an Organ‐on‐a‐Chip

**DOI:** 10.1002/advs.202103241

**Published:** 2022-03-14

**Authors:** Girija Goyal, Pranav Prabhala, Gautam Mahajan, Bruce Bausk, Tal Gilboa, Liangxia Xie, Yunhao Zhai, Roey Lazarovits, Adam Mansour, Min Sun Kim, Aditya Patil, Danielle Curran, Jaclyn M. Long, Sanjay Sharma, Abidemi Junaid, Limor Cohen, Thomas C. Ferrante, Oren Levy, Rachelle Prantil‐Baun, David R. Walt, Donald E. Ingber

**Affiliations:** ^1^ Wyss Institute for Biologically Inspired Engineering at Harvard University Boston MA 02115 USA; ^2^ Department of Pathology Brigham and Women's Hospital and Harvard Medical School Boston MA 02115 USA; ^3^ Vascular Biology Program and Department of Surgery Boston Children's Hospital and Harvard Medical School Boston MA 02115 USA; ^4^ Harvard John A. Paulson School of Engineering and Applied Sciences Harvard University Cambridge MA 02139 USA

**Keywords:** antibody, germinal center, lymph node, organ chip, vaccine

## Abstract

Lymphoid follicles (LFs) are responsible for generation of adaptive immune responses in secondary lymphoid organs and form ectopically during chronic inflammation. A human model of ectopic LF formation will provide a tool to understand LF development and an alternative to non‐human primates for preclinical evaluation of vaccines. Here, it is shown that primary human blood B‐ and T‐lymphocytes autonomously assemble into ectopic LFs when cultured in a 3D extracellular matrix gel within one channel of a two‐channel organ‐on‐a‐chip microfluidic device. Superfusion via a parallel channel separated by a microporous membrane is required for LF formation and prevents lymphocyte autoactivation. These germinal center‐like LFs contain B cells expressing Activation‐Induced Cytidine Deaminase and exhibit plasma cell differentiation upon activation. To explore their utility for seasonal vaccine testing, autologous monocyte‐derived dendritic cells are integrated into LF Chips. The human LF chips demonstrate improved antibody responses to split virion influenza vaccination compared to 2D cultures, which are enhanced by a squalene‐in‐water emulsion adjuvant, and this is accompanied by increases in LF size and number. When inoculated with commercial influenza vaccine, plasma cell formation and production of anti‐hemagglutinin IgG are observed, as well as secretion of cytokines similar to vaccinated humans over clinically relevant timescales.

## Graphical abstract

Primary human lymphocytes taken from blood are reprogrammed into lymphoid follicles (LF) by superfusion in an organ chip device. These ectopic LFs express AID and can be induced to form plasma cells and antigen‐specific antibodies upon activation with different stimuli including adjuvants and seasonal influenza vaccines.

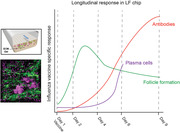

## 1 Introduction

New vaccines and immunotherapies are currently evaluated in animal models, which can lead to unpredicted toxicities or poor efficacy in clinical trials because of species‐specific differences in immune responses.^[^ ^1^ ^]^ Preclinical experiments can be conducted in vitro using human immune cells collected from blood; however, even these results often fail to predict patient responses.^[^ ^2^, ^3^ ^]^ One major reason for this failure is that in vivo immune responses commonly occur within the highly specialized tissue microenvironment of lymphoid follicles (LFs) that normally exist within the cortex of secondary lymphoid organs such as lymph nodes. LFs can form ectopically (in other organs) as a result of inflammation where they are similarly responsible for generating an adaptive immune response.^[^ ^4^ ^]^ Importantly, DNA modifying enzymes required by B cells to switch from one antibody (Ab) isotype to another, such as Activation‐Induced Cytidine Deaminase (AID), are only expressed by B cells resident within LFs.^[^ ^5^ ^]^ Upon immune activation by antigen, helper T cells in the LF enable the B cells to switch isotypes and produce high‐affinity antibodies (Abs); when activated in this manner, LFs are called germinal centers.^[^ ^6^ ^]^ The importance of the 3D tissue niche is made clear by the observation that class switching is defective in mice or humans that lack lymph nodes, and this leads to recurrent infections and morbidity even though B‐lymphocytes from these animals or patients can still be induced to undergo class switching in vitro.^[^ ^7^ ^]^

Thus, creation of a well‐defined experimental model that can recapitulate ectopic human LF formation in vitro could provide insight into the development of these structures that are so critical for immune responses. Given the current challenges related to the availability of animal models, and particularly non‐human primates, for testing vaccines for COVID‐19 and other diseases,^[^ ^8^ ^]^ development of an all‐human ectopic LF model using easily accessible cell sources also could provide a valuable preclinical tool for evaluation of vaccines and adjuvants.

Human organ‐on‐a‐chip (Organ Chip) microfluidic culture technology has been previously used to recapitulate both normal physiology and disease states of multiple human organs (e.g., lung, intestine, kidney, bone marrow, brain, etc.).^[^ ^9^ ^]^ These complex microphysiological systems enable application of dynamic fluid flow and 3D architecture, as well as control of cellular and extracellular matrix (ECM) and cellular composition, which are key to developmental regulation and tissue function. Thus, in the present study, we set out to determine whether a previously described Organ Chip device that contains two parallel channels separated by a microporous membrane^[^ ^10^ ^]^ can be leveraged to meet this challenge. As human cell sourcing is always a challenge, we isolated primary human B and T lymphocytes non‐invasively from peripheral blood for these studies. Here, we show that primary blood‐derived B and T lymphocytes from multiple human donors spontaneously self‐assemble into ectopic LFs that express AID and CXCL13 and support plasma cell differentiation when cultured within an ECM gel contained in one channel of this two‐channel Organ Chip while being fed by medium flowed through the other parallel channel. These studies revealed that this microfluidic form of superfusion culture both supports ectopic LF formation and prevents autoactivation previously reported in high‐density cultures of human B cells.^[^ ^11^ ^]^ Human LF Chips additionally containing autologous dendritic cells (DCs) display antigen‐specific IgG production as well as secretion of clinically relevant cytokines when vaccinated with a commercially available, split virion trivalent influenza vaccine, and this same model is able to detect immune‐enhancing activity of a squalene‐in‐water emulsion (SWE) adjuvant that could have great value for vaccination in low resource nations.

## 2 Results

### 2.1 Self‐Assembly of T and B Lymphocytes into LFs on‐Chip with Minimal Autoactivation

**Figure 1:**
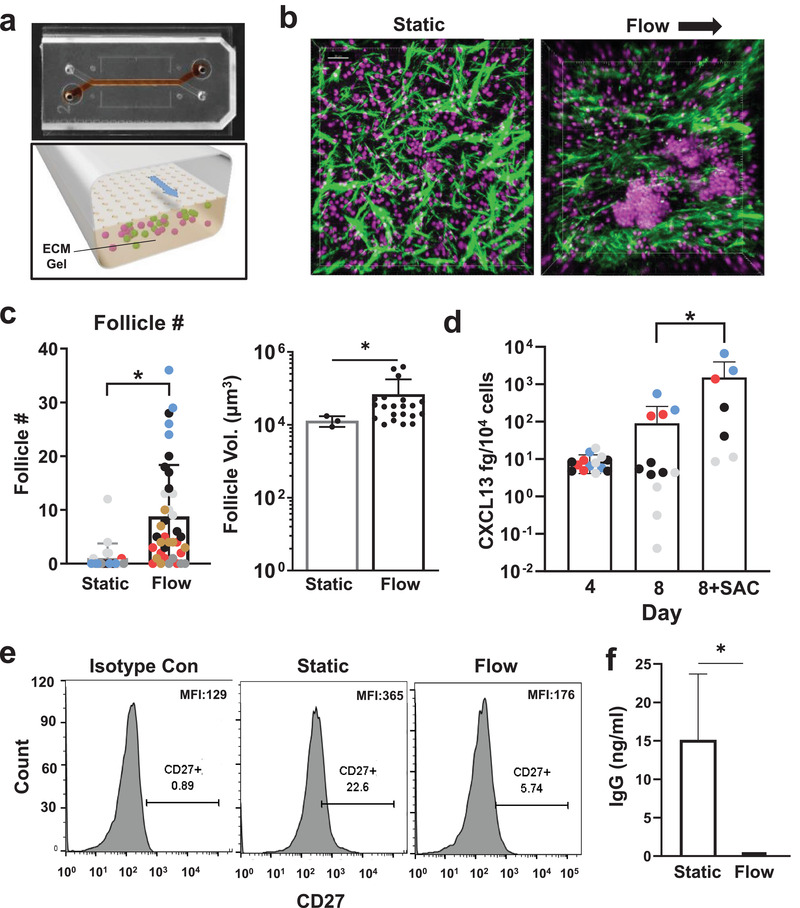
Perfusion induces follicle formation in the human LF chip. a) Photograph (top) of the 2‐channel Organ Chip device used to create the human LF Chip with red dye filling the lower microchannel and a schematic of a cross‐section of the device (bottom) showing how the lower channel is filled with an ECM gel containing human lymphocytes, which are fed through the porous membrane separating the channels by medium that is flowed through the upper channel. b) Second harmonic images of ECM fibrils (green) combined with fluorescence images of Hoechst stained nuclei (magenta) of human lymphocytes growing at high density within ECM gels maintained within the lower channel of the LF Chip either under static conditions (Static) or with dynamical perfusion (Flow; arrow indicates direction) (bar, 30 µm). c) Quantification of LF number (Follicle #) and volume (Follicle Vol.) in 3D ECM gels that are cultured statically (Static) or with active perfusion (Flow) in Organ Chips for 4 days. Different colored data points indicate different donors (*n* = 6) and each point represents follicle numbers (left) per field of view from >2 chips. The volume for each follicle observed under Static or Flow is reported for one representative donor (right). Similar differences in follicle size are observed in two additional donors. Error bars indicate standard deviation (SD); *, *p* < 0.05 using an unpaired Student's *t*‐test with Welch's correction. d) Secreted CXCL13 protein detected within the effluent of the LF Chip cultured for 4 or 8 days in the presence or absence of SAC antigen using a Simoa assay. Different colored data points indicate different donors (*n* = 4) and each point represents an individual chip. Error bars indicate SD; *, *p* < 0.05 using one way ANOVA is conducted to identify any significant differences followed by the Fisher's LSD test. e) CD27 expression on B cells assessed by flow cytometry in isotype controls (left), static culture (middle), and the perfused LF chip (right). Representative results displayed 1 chip from one donor, and similar results are obtained with 2 chips using cells from two different donors; the percentage of cells in the positive peak is indicated below CD27+ in each graph. MFI is shown at the top left of the histograms. f) IgG levels determined by ELISA (limit of detection = 49 pg mL^−1^) and normalized for culture volume in static culture (Static) versus perfused LF chips (Flow) in 2 chips each from 2 donors (*n* = 4). Error bars indicate SD; *p < 0.05 using an unpaired Student's *t*‐test with Welch's correction.

We used a microfluidic Organ Chip containing two channels separated by a porous membrane^[^ ^10^ ^]^ in which human T and B lymphocytes isolated from blood apheresis collars were cultured at a high density (1.5–2 × 10^8^ cells mL^−1^; 1:1 ratio) within an ECM gel composed of Matrigel and type I collagen. The gel‐filled the lower channel of the device and was supplied with oxygen and nutrients through continuous perfusion of culture medium through the upper channel (**Figure** **1**a; Figure S1a, Supporting Information). We chose this high culture density because high‐density lymphocyte suspension cultures have been shown to be more predictive of human clinical responses than lower density cultures.^[^ ^12^ ^]^ Human T and B lymphocytes commonly only form small cell aggregates when co‐cultured in suspension or on membranes,^[^ ^13^ ^]^ and we observed similar behavior when we cultured unstimulated, patient‐derived T and B cells at high density under static conditions in the same ECM gel (Figure 1b, Figure S2, Supporting Information). In contrast, when these patient‐derived T and B‐lymphocytes were cultured under superfusion on‐chip for 3–4 days, they spontaneously self‐assembled into much larger 3D multicellular aggregates resembling small LFs (Figure 1b, Figure S1b, Supporting Information).

Second‐harmonic microscopic imaging of these cultures revealed that dynamic medium flow through the parallel channel of the chip resulted in realignment of matrix fibrils within the ECM gel that aligned with the flow direction whereas the fibrils formed larger aggregates and were oriented randomly when the lymphocytes were cultured in the same ECM gel under static conditions (Figure 1b). Interestingly, this structural reorganization of the ECM was accompanied by a significant increase in the number and size of the LFs compared to static 3D ECM gels after 3 days of culture when analyzed in chips created with cells from multiple different human donors (Figure 1b‐d; Figure S1c,d, Supporting Information). In the static 3D cultures, LF formation was only observed in ≈25% (1 of 4) of the human donors, whereas 100% (6 out of 6) of the donors formed LFs in perfused microfluidic chip cultures (Figure 1c), and these were also significantly larger in volume (Figure 1d) indicating a distinct and additional role of superfusion as compared to the ECM composition or mechanics alone. The total number of LFs did not increase beyond 4 days and appeared to slightly decrease by 7 days of culture in the absence of antigenic activation (Figure S1d, Supporting Information). T and B cells also were found to be in close contact within the ectopic follicles, forming cell‐cell contacts on‐chip with polarized expression of the T cell co‐receptor CD3 (Figure S1e, Supporting Information). Culture of lymphocytes on ECM‐coated plates induces a similar tissue‐like polarization in circulating T cells.^[^ ^12^, ^14^ ^]^ Similarly, we found T cell polarization to be significantly increased in the LF Chips containing cells in 3D ECM gels whether maintained under static or perfused conditions compared to cells in conventional planar 2D culture lacking any ECM (Figures S1e and S2, Supporting Information).

Lymph nodes are formed because of a positive feedback loop between lymphoid tissue inducer and organizer cells leading to production of CXCL13, CCL19, and CCL21 chemokines that promote recruitment of lymphocytes.^[^ ^15^ ^]^ While the cellular and molecular cues that lead to LF formation are not fully defined, CXCL13 is key requirement for LF assembly^[^ ^16^ ^]^ and it is often used as a biomarker of LF formation.^[^ ^4^ ^]^ CXCL13 levels in plasma also correlate with both LF formation and vaccine responses in humans.^[^ ^16^, ^17^ ^]^ Moreover, CXCL13 and its receptor CXCR5 are key genes in the transcriptomic signature exhibited by tertiary lymphoid organs, which are ectopic LFs forming outside secondary lymphoid organs.^[^ ^4^ ^]^ Importantly, we found that the human lymphocytes produce CXCL13 under baseline conditions when cultured for 4 to 8 days on‐chip, and its expression increased significantly by day 8 when the LF Chips were exposed to the bacterial *S. aureus* Cowan I (SAC) antigen (Figure 1d). In contrast, T and B cells from 4 independent donors in conventional planar 2D culture lacking any ECM did not secrete detectable CXCL13 after 4 days (limit of detection = 12 fg mL^−1^). CXCL13 was also expressed in static 3D cultures suggesting that either high cell density, 3D culture, or contact with the ECM gel were causing the cells to produce CXCL13 (Figure S1f, Supporting Information).

Autoactivation of B cells has been reported to be a challenge in high density cultures,^[^ ^11^ ^]^ and so we expected that the increased numbers of cell‐cell contacts we observed in our LF chips might be leading to autoactivation. Surprisingly, however, we did not observe significant autoactivation of B cells in our perfused LF chips (Flow), even though the same cells became autoactivated when cultured at this high density without perfusion (Static), as seen by an increased percentage of cells that express CD27 compared to cells cultured on‐chip under flow or an isotype control (22.6% versus 5.7% versus 0.9%, respectively) (Figure 1e). To further assess autoactivation, naive B cells isolated from blood apheresis collars using CD27 depletion (Figure S3a, Supporting Information) were combined with T cells and on organ chip devices with (Flow) or without perfusion (Static). We observed spontaneous class switching in static cultures as seen by production of high levels of IgG (Figure 1f), which was again consistent with the cells autoactivating. Importantly, however, we did not observe any class switching in the perfused LF chips under these baseline conditions as indicated by the undetectable levels of IgG. (Figure 1f). Similarly, IgM levels were extremely high in static cultures, but not in the perfused LF chips (Figure S3b, Supporting Information). Reduction to undetectable levels of IgG and IgM cannot be explained by the culture volume of the LF chip (2 mL) as it is only 8 times higher than that in the static 3D culture (250 µL).

### 2.2 Ectopic LFs Upregulate AID Expression

**Figure 2:**
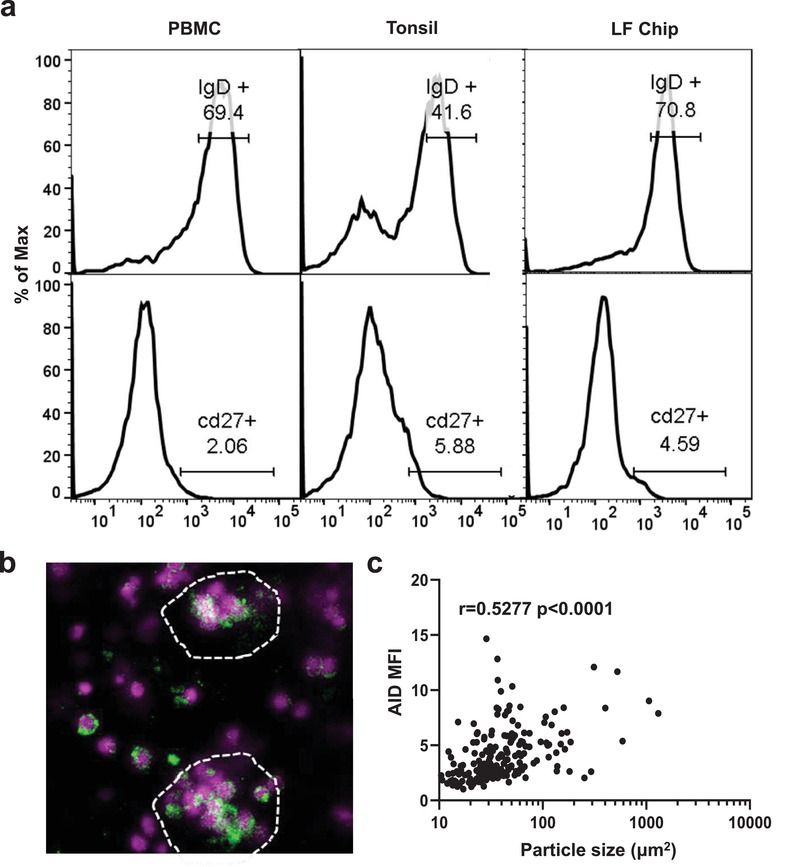
B cells remain quiescent yet express AID in LFs formed on‐chip. a) Representative flow cytometric characterization of B cells stained for IgD and CD27 in the initial PBMC sample (PBMC; *n* = 3) compared with cells from explanted tonsils (Tonsil; *n* = 2), or cells cultured in the LF chip for 4 days (LF Chip; *n* = 3). The percentage of cells in the positive peak is indicated above the gate drawn on the histogram. b) Representative confocal immunofluorescence micrograph showing AID expression (green) in B cells cultured in the LF chip for 4 days (similar results are obtained with 4 donors); Hoechst stained nuclei in the lymphocytes are shown in blue. c) Quantification of AID expression levels in 183 particles ranging from single cell sized regions‐of‐interest to large follicles from 4 independent fields of view measured as MFI plotted as a function of particle size (Particle area) in perfused Organ Chips cultured for 4 days. Each data point represents 1 particle and particles from 4 images from the LF chips created from one representative donor shown. Similar results obtained in 3 additional donors. Non‐parametric Spearman correlation shown.

B cells in the blood are a minor population of peripheral blood mononuclear cells (PBMCs) compared to T lymphocytes, and they are mostly naïve with about 70% appearing as IgD^+^ CD27^−^ cells when analyzed by FACS analysis, while tonsils that contain multiple LFs have a high number of B cells, but only about 40% of them are naive IgD^+^ CD27^−^ cells^[^ ^18^, ^19^ ^]^ (**Figure** **2**a). Importantly, the unstimulated B cells within the LF Chips retained the phenotype of PBMCs, again with about 70% of cells appearing IgD^+^ CD27^−^ (Figure 2a).

Surprisingly, despite retaining the IgD and CD27 levels of freshly isolated PBMCs, B cells within the ectopic LFs that self‐assembled on‐chip were induced to express the AID enzyme which was absent in both PBMCs and 2D lymphocyte cultures (Figure 2b and Figure S4b, Supporting Information), indicating acquisition of follicular functions that are not normally present in circulating B cells.^[^ ^20^, ^21^, ^22^ ^]^ AID expression is important because it mediates the critical Ab class switching response within germinal centers in the lymph node in vivo.^[^ ^5^ ^]^ We detected AID expression in B cells even in static 3D culture again suggesting a contribution of the ECM, 3D culture, and/or high cell density (Figure 2b; Figure S4a, Supporting Information). However, when we quantified the mean fluorescence intensity (MFI) of lymphocytes, we found that AID expression increased linearly with follicle size, with single cells having the lowest MFI (Figure 2c). We independently estimated the number of AID^+^ B cells within LFs by measuring the percentage of the follicle area that contained AID^+^ cells and found that more AID+ cells participate in follicles as follicle size increases (non‐parametric Spearman *r* value = 0.3290 and *p* = 0.0024) (Figure S4c, Supporting Information). Lymphocytes cultured within the perfused Organ Chips also exhibited greater AID expression as compared to static 3D cultures (Figure S4d, Supporting Information).

Flow cytometry was used to compare the number of B cells expressing IgM or IgD, follicular T helper (Tfh) cells (as defined by co‐expression of both CXCR5 and PD‐1), and CXCR5 expression in B cells in the LF Chips compared to conventional 2D culture in a plastic dish (Figure S5a, Supporting Information). IgM^+^ cells in the LF Chips are composed mainly of naive B cells as indicated by the lack of CD27,^[^ ^23^ ^]^ whereas the IgG^+^ cells represent a small population of previously activated, class switched memory cells. Given the expected variability between the 4 human lymphocyte donors tested, this analysis did not show any statistically significant differences in the total number of B cells or Tfh cells between 2D cultures and the human LF chips; however, LF Chips created with cells from all 4 donors had increase numbers of IgM+B cells (Figure S5b, Supporting Information). Flow cytometric analyses also revealed that 82% of the B cells in the unstimulated LF chips express CXCR5 that is the receptor for CXCL13, and a fraction of the CD4 T cells are also CXCR5^+^ (Figure S5a, Supporting Information). Thus, these surface molecules represent potential cellular targets for secreted CXCL13 that is produced in the human LF Chips.

### 2.3 Class Switching and Plasma Cell Differentiation

**Figure 3:**
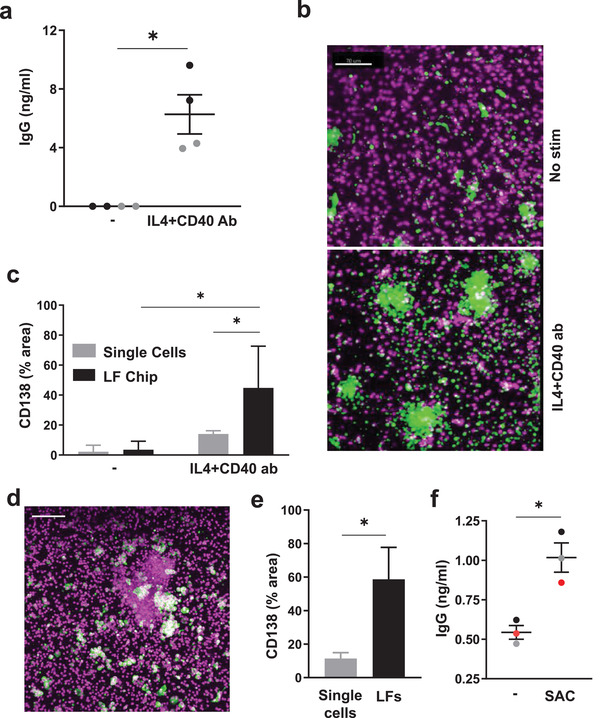
B cells exhibit class switching and undergo plasma cell formation in the LF chip. a) Total IgG production measured in the effluents of LF chips when engineered with naïve B cells and bulk T cells after 6 days culture in the presence or absence (‐) of IL4 and anti‐CD40 Ab Each dot indicates results from an individual chip (*n* = 2) created with cells from two donors (black and gray); *, *p* < 0.05 using an unpaired Student's *t*‐test with Welch's correction. b) Immunofluorescence micrographs showing cells in unstimulated LF chips (No stim.) or chips treated with IL4 and anti‐CD40 Ab stained for CD138 (green) and nuclei (magenta); similar results are obtained with cells from 3 different donors. c) Quantification of CD138 expression in single cells (gray bars) versus cells located within LFs (black bars) in the same LF chips. Error bars indicate SD based on analysis of 5 randomly selected fields from 1 donor, and similar results are obtained with LF Chips containing cells from 3 different donors. *, *p* < 0.05 using a two way ANOVA to identify any significant differences followed by the Fisher's LSD test. d) Immunostaining for CD138 (green) and nuclei (magenta) in SAC‐treated LF Chips (similar results obtained with 3 donors; bar 100 µm). e) CD138 levels measured as a % of projected area labeled for CD138 in lone cells (Single Cells) versus cells in follicles (LFs) within ECM gels in perfused Organ Chips. Results shown are from 5 randomly selected fields from 1 LF Chip created with cells from one donor, and similar results are obtained with cells from 3 different donors. Error bars indicate SD; * *p* < 0.05 using an unpaired Student's *t*‐test with Welch's correction. f) Total IgG levels measured in the effluent of LF Chips 3 days after treatment with SAC. Each colored dot indicates results from one chip from each of 3 different donors. Error bars indicate standard error of mean (SEM); *p* < 0.05 using an unpaired Student's *t*‐test.

Naive B cells stimulated with IL4 and anti‐CD40 Ab have been shown to undergo Ab class switching,^[^ ^24^ ^]^ and thus, we used these stimuli to further characterize B cell functionality in the LF chips. When we perfused the LF chips with IL‐4 and this CD40 ligand, IgG was detected in the chip outflows whereas it was not detected in unstimulated chips (**Figure** **3**a), confirming that B cells are functional and capable of class switching in these Organ Chip cultures. Importantly, we also observed formation of CD138^+^ plasma cells and their organization within large clusters in many follicles on day 7 in the stimulated LF chips (Figure 3b). While CD138 expression could be detected in some single cells, again it was preferentially expressed by cells that clustered within the self‐assembled ectopic LFs (Figure 3c), thus mimicking plasma cell development and differentiation that occur in LFs in vivo.^[^ ^25^, ^26^ ^]^

As LFs can be induced to form as a result of viral^[^ ^27^ ^]^ or bacterial^[^ ^28^ ^]^ infections, we next explored if these ectopic LF Chips can be used as preclinical tools to study adaptive immune responses to pathogens by using the SAC antigen to mimic the presence of dead bacteria. Exposure to this bacterial antigen again induced CD138^+^ plasma cell formation to a much greater degree in the LFs that formed on‐chip compared to nearby single cells (Figure 3d,e), and importantly, this was accompanied by increased production of IgG (Figure 3f). The LF chip, therefore, provides a way to model LFs that self‐assemble, form plasma cells, undergo class switching, and generate polyclonal IgG Abs in response to bacterial antigens.

### 2.4 The LF Chip Recapitulates Human Seasonal Vaccination and Adjuvant Responses

Existing preclinical models used for testing of vaccines involve 2D cultures or animal models that do not faithfully mimic human responses. Even more concerning is that the animal model that is closest to human—non‐human primates—is currently in short supply and encumbered by ethical concerns.^[^ ^8^ ^]^ Protective immunity induced by vaccination requires plasma cell production of antigen‐specific antibodies, and vaccination‐induced formation of ectopic LFs can contribute to this response.^[^ ^29^ ^]^ Thus, we next explored the human LF Chip that can be used as study human vaccine responses.

As vaccine antigens are presented by DCs in the draining lymph node in vivo, we differentiated autologous human DCs from monocytes and included them along with B and T cells as 2% of the total population. There were reports in 1970–1980 suggesting that influenza vaccination can be performed in vitro using peripheral blood‐derived 2D cultures; however, researchers now agree that these responders likely had a recent exposure to influenza and that conventional 2D cultures of PBMC are poor at producing a recall antibody response to influenza vaccination. To explore the utility of the human LF Chip as a preclinical human in vitro testing platform that can be used to derisk adjuvants as well as vaccines, we prepared 2D cultures and LF Chips from the same cellular pool containing an equal ratio of T and B cells with 2% DCs (Figure S6a, Supporting Information). As bulk B cells were used as in these experiments, the results do not distinguish between naïve and memory responses. Instead, they are representative of the clinical response to seasonal influenza vaccines as the majority of the lymphocyte donors have been previously exposed to natural influenza infection or influenza vaccines. Responses of the 2D cultures and LF chips to vaccination with split virion influenza alone or combined with SWE adjuvant^[^ ^30^ ^]^ were analyzed using cells from 4 separate donors.

We found that the sensitivity of commercially available plate‐based ELISA methods and hemagglutination inhibition assays commonly used to detect anti‐influenza hemagglutinin (HA) antibodies in human serum is too low for in vitro studies. To overcome this limitation, we modified a high sensitivity digital ELISA assay^[^ ^31^ ^]^ to detect influenza virus strain‐specific antibodies. As in vitro positive controls, we analyzed 2D cultures of cells obtained from human tonsils stimulated with split virion trivalent influenza vaccine based on previous studies,^[^ ^32^ ^]^ which confirmed that the digital ELISA could detect high levels of anti‐HA antibodies in one of the two tonsil donors (Figure S6b, Supporting Information).

**Figure 4:**
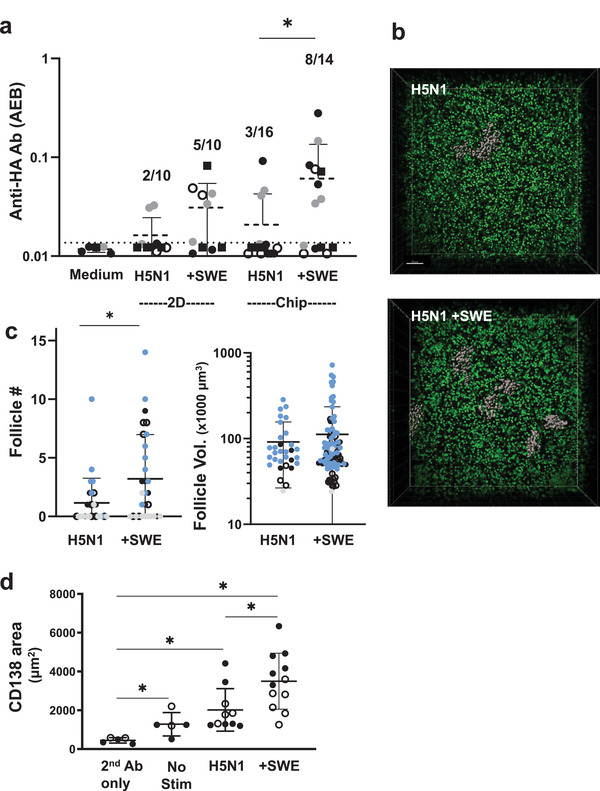
Vaccine and adjuvant‐induced Ab production and follicle formation in the human LF chip. a) Anti‐HA Ab signal detected in the effluents of the LF Chips (Chip) or in the medium of cells maintained in 2D culture (2D) for 9 days using the Simoa digital ELISA when unstimulated (Medium) or stimulated with H5N1 antigen alone (H5N1) or with SWE (+SWE). Each data point indicates one well or chip; different colored points indicate chips or wells from 4 independent donors. The fraction of total samples that exhibited anti‐HA Ab signal >LOD is indicated in the text above the points. Simoa values are presented as average enzyme per bead (AEB) and error bars indicate SD. Statistical differences are tested using the Kruskal‐Wallis test followed by pair‐wise testing using the uncorrected Dunn's test); *p* < 0.05. b) A 3D confocal microscopic stack view showing pseudocolored follicles (grey) and cell nuclei (green) present within ECM gels cultured for 3 days within a perfused LF Chip when vaccinated with split H5N1 influenza antigen (H5N1) in the absence or presence of SWE adjuvant (H5N1 + SWE); bar, 50 µm. c) Graph showing quantification of the number (left) and size (right) of follicles observed in LF Chips generated with cells from 4 different donors indicated by different colored symbols. Each data point indicates one field of view (follicle #, *n* > 26) or one individual follicle (follicle vol., *n* > 30); error bars indicate SD; *, p < 0.05 using an unpaired Student's *t*‐test with Welch's correction. d) Graph showing quantification of the CD138+ area observed in LF Chips generated with cells from two different donors (open and filled squares); Each data point indicates one field of view; * *p* < 0.05, using a Brown‐Forsythe one way ANOVA followed by unpaired *t*‐test with Welch's correction for unequal variances.

When we used this more sensitive assay, we found that when vaccinated with split influenza H5N1, 2D cultures and human LF chips only displayed limited induction of anti‐HA Ab production with only 1/4 and 2/4 donors showing this response, respectively, and this was only observed in some of the culture replicates (**Figure** **4**a, Figure S6c, Supporting Information). However, the addition of the SWE adjuvant increased both the levels of secreted anti‐HA Ab as well as the number of replicate 2D wells or chips responding. 3/4 donors showed some anti‐HA Ab in 2D cultures with SWE and all 4 donors had at least one positive Ab producing LF Chip with SWE. However, only the human LF chips vaccinated with split H5N1 antigen and SWE displayed statistically significant improvement over unadjuvanted controls. This enhancement in Ab responses also was accompanied by an increase in LF number in 4 donors tested suggesting that increased LF formation correlates with better Ab responses in vitro (Figure 4b,c). Moreover, a greater number of LFs were maintained in culture at 9 days after vaccination with SWE adjuvant compared to antigen alone (Figure S7, Supporting Information). Plasma cell differentiation was assessed by CD138 staining and quantification of the total area covered by CD138+ cells in each field of view at days 3 and 5 after vaccination. While negligible CD138 staining was detected at day 3 (not shown), split H5N1 leads to a modest increase in plasma cell number at day 5, and when it was administered in combination with the SWE adjuvant, there was a significant enhancement in the number of CD138+ plasma cells (Figure 4d). Importantly, this is the same time when circulating plasma cells can first be detected in the blood of vaccinated individuals.^[^ ^33^ ^]^ Taken together, these studies show that the human LF Chip is more effective at revealing the effect of SWE on LF formation and antigen‐specific Ab production in response to seasonal vaccination than conventional 2D cultures.

**Figure 5:**
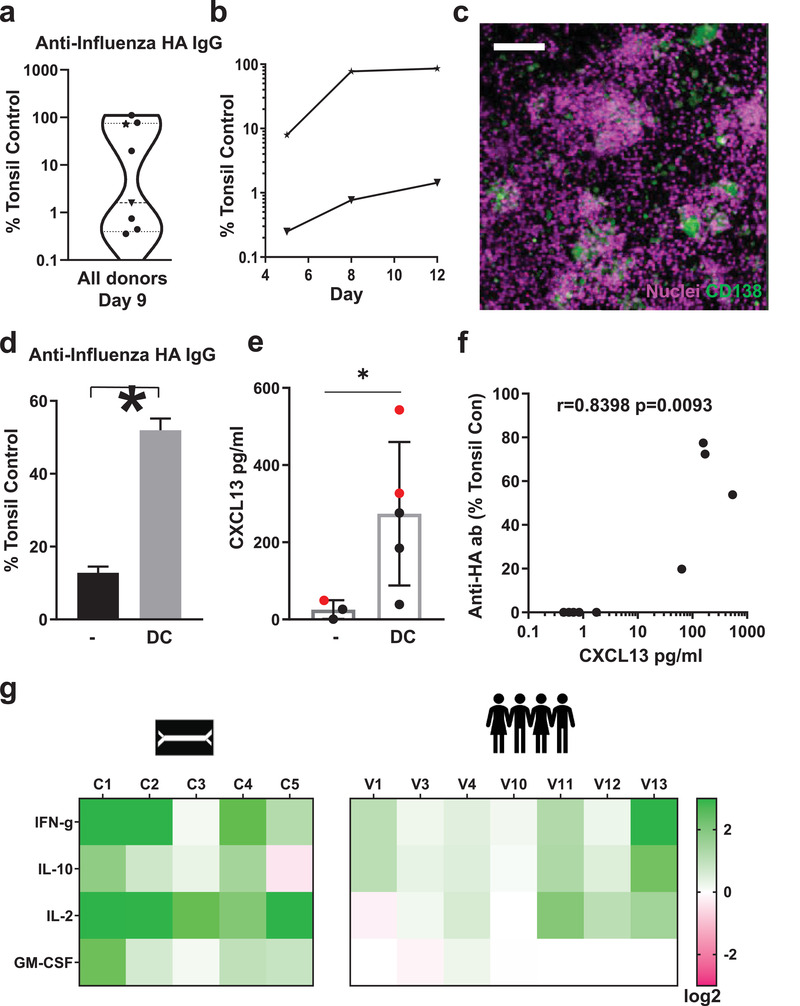
Influenza vaccination in vitro in the human LF chip. a) Violin plot of anti‐HA IgG levels that are specific to the Brisbane 59 H1N1 strain (Anti‐HA IgG) in the effluent of LF Chips 9 days after vaccination with Fluzone presented relative to levels measured in a vaccinated culture of tonsillar cells (Tonsillar Control), as detected using a digital ELISA. Each data point represents average of 2–6 chips created with cells from one donor (total 8 donors tested). b) Time course of anti‐HA IgG secretion measured in chip effluents over 5 to 12 days of culture using LF Chips containing cells from representative donors from the high (★) and low (▼) Ab producer groups shown in a. c) Immunofluorescence micrograph showing CD138 staining (green) in a Fluzone‐stimulated LF Chip containing cells (nuclei, magenta) from a high anti‐HA Ab producer (similar results are obtained with 3 different high Ab producer donors; bar, 100 µm). d) Anti‐HA Ab levels that are specific to the Brisbane 59 H1N1 strain in the effluent of LF Chips with or without DCs, 9 days after vaccination, as detected by a digital ELISA, presented relative to levels measured in a culture of tonsillar cells. Mean levels from 3 replicate measurements from one chip generated from one donor are shown, and similar results are obtained in LF Chips created with cells from two different donors. Error bars indicate SD; *, *p* < 0.05 using an unpaired Student's t‐test with Welch's correction. e) CXCL13 levels in the effluent of the LF chip with or without DCs, 5 days after vaccination, as detected by a digital ELISA. Each colored dot represents one chip with cells from two different donors (black and red dots, *n* ≥ 3). Error bars indicate SD; *, *p* < 0.05 using an unpaired Student's *t*‐test with Welch's correction. f) Levels of anti‐HA Ab specific to the Brisbane 59 H1N1 strain in the effluent of LF chips at day 9 plotted relative to CXCL13 levels 5 days after vaccination. Results from 5 donors are shown with each dot representing an individual chip (*n* = 9); an analysis of the non‐parametric Spearman correlation between CXCL13 and IgG levels are in Graphpad Prism is shown. g) Heat map of the fold change (Log_2_) in the levels of cytokines (IFN‐*γ*, IL‐10, IL‐2, GM‐CSF) measured using a digital ELISA in effluents of LF chips generated with cells from 5 different donors, 3 days after vaccination (C1‐5) compared to unvaccinated chips, and to levels measured in peripheral blood from 7 individuals (V1, 3, 4, 10–13) 1 day after vaccination as compared to their prevaccination levels.

Finally, we explored whether we could generate a clinically relevant vaccination response in the LF Chips by inoculating them with commercial Fluzone influenza vaccine which contains 3 different strains of virus. Using this method, we found that LF Chips generated with cells from 8 different human donors all produced anti‐HA antibodies at detectable levels when compared to supernatants of tonsil explant cultures that were highly positive for anti‐HA antibodies after Fluzone vaccination. We detected two patient subsets when we compared anti‐HA IgG levels in human LF chip effluents to Ab levels present in the responsive vaccinated tonsil supernatant (**Figure** **5**a, Figure S6b, Supporting Information). LF Chips from one patient group responded by producing similar levels of anti‐HA IgG in the LF Chip as in the tonsil preparation whereas the other were low responders (Figure 5a); these different types of responses could be a result of previous influenza exposure, varying levels of precursor frequency, or other factors. Abs were first detectable in the chip effluents about 5 days after immunization and their levels increased significantly over the following week in both the low and high responder groups (Figure 5b). Plasma cells also were detected at 5 days after immunization in the vaccinated LF Chips made with cells from donors that exhibited high levels of Ab production (Figure 5c). Also, as expected based on past work showing the importance of antigen‐presenting DCs for generation of a vaccination response,^[^ ^34^ ^]^ there was a significant reduction in levels of anti‐influenza HA‐specific IgG (Figure 5d) and CXCL13 (Figure 5e) when DCs were not included in the LF chips. Interestingly, levels of CXCL13 measured on‐chip 5 days post‐vaccination were also highly predictive of anti‐influenza Ab responses measured at day 9 when compared across chips from 5 donors (Figure 5f).

Finally, we quantified levels of four cytokines within the effluent of the vaccinated LF chips that are important for T cell expansion, survival, and helper functions (IFN‐*γ*, IL10, IL2, GM‐CSF), and compared their levels to those measured in the serum previously obtained in a study of human volunteers vaccinated with influenza vaccines. Serum was collected from volunteers before and one day after vaccination and frozen for further analyses. These studies showed IFN‐*γ*, and IL‐2 increased up to eightfold in the human LF chip 4 days after vaccinating with Fluzone and IL‐10 increased up to fourfold depending on the donor (5 donors, C1‐C5). Serum from human volunteers also showed similar (up to 4‐fold) upregulation of these 3 cytokines across 7 individuals (Figure 5g). GM‐CSF levels were also increased in the human LF chip; however, its levels did not change after vaccination in the human serum.

## 3 Discussion

Animal models are the gold standard for advancing vaccine candidates and immunotherapeutics to the clinic. However, because the immune systems of animals are significantly different from humans, unpredicted toxicities or poor efficacies have resulted when vaccines and immunotherapies entered clinical trials.^[^ ^1^ ^]^ For example, the severe cytokine syndrome that developed in patients enrolled in the Phase I clinical trial of the CD28 superagonist TGN1412 had not been detected in preclinical animal models.^[^ ^2^ ^]^ High density cultures of PBMCs were later shown to predict high levels of cytokine release by TGN1412; however, it is not possible to use these static, high density 2D cultures of PBMC to study how immune responses induce reorganization of lymphocytes in 3D that leads to LF formation and expansion, as well as associated Ab class switching, plasma cell formation, germinal center organization, and secretion of high‐affinity Abs. Humanized mice have been explored as a possible alternative to study vaccines in vivo, but they are severely immunocompromised, require use of human fetal tissues which has ethical implications, and they lack lymph nodes, which causes defects in class switching, affinity maturation, and long‐lived plasma cell formation.^[^ ^35^ ^]^ Most concerning is that during the current COVID19 pandemic, non‐human primates that have been used to test vaccines have become a scarce resource,^[^ ^8^ ^]^ which could limit development of newer vaccines that might be required as new variants emerge.

In this study, we described microfluidic Organ Chips containing primary human B and T cells isolated non‐invasively from peripheral blood that spontaneously self‐assemble into LFs that undergo Ab class switching, form plasma cell clusters, and secrete antigen‐specific IgG when stimulated with antigen in the presence of DCs. We found that they can be used to evaluate the efficacy of seasonal vaccines as well as adjuvants. This was demonstrated by vaccinating the LF chips in vitro using a trivalent commercial influenza vaccine and a split H5N1 (A/turkey/Turkey/1/2005) pandemic influenza antigen formulated with SWE, a squalene oil‐in‐water adjuvant.

It is important to note that others have studied cultured human tonsillar lymphocytes obtained from patients^[^ ^36^, ^37^ ^]^ or created putative 3D models of the lymph node^[^ ^38^, ^39^ ^]^ to conduct preclinical testing of vaccines. However, these experimental systems do not demonstrate lymph node‐like biomarkers, *de novo* LF formation, or survival of plasma cells, and hence, cannot be used to study LF formation, physiology, or the underlying basis of human adaptive immune responses in vitro as the LF Chip can.^[^ ^40^ ^]^

The formation of germinal centers is a hallmark of protective immunity against infectious agents and formation of germinal center‐like structures in ectopic locations can predict vaccine^[^ ^27^, ^29^ ^]^ and immunotherapy^[^ ^4^, ^41^ ^]^ efficacy. However, ectopic LF formation also can result from pathological activation of the immune system, as observed in patients with autoimmunity.^[^ ^42^, ^43^ ^]^ There is currently no model of human LF formation where this process can be studied under controlled conditions in vitro. There was a recent report describing self‐aggregation of tonsillocytes in static cultures^[^ ^40^ ^]^; however, tonsillar (and splenic) tissue is difficult for most labs to access as surgical intervention is required and visualization of higher order multicellular organization is difficult using this approach. Further, the effect of cell density on autoactivation and assay readout remains to be investigated. In contrast, the in vitro Organ Chip approach we describe here results in self‐assembly of LFs from peripheral blood‐derived circulating immune cells, which are much easier to obtain. This is a major advantage of the LF Chip that is of particular value when trying to scale up this approach for preclinical testing of vaccines, for example.

Importantly, because we are able to control all components of the model and were able to observe LF formation over time in vitro with Matrigel providing the only exogenous growth factors and no additional stromal or myeloid cells, we also were able to gain new insights into how follicle assembly is controlled. First, we were able to clearly demonstrate that the presence of B and T lymphocytes is sufficient to induce LF formation, which is consistent with recent studies of tertiary lymphoid organ formation.^[^ ^44^, ^45^ ^]^ In addition, we discovered that superfusion promotes LF formation, which appears to be mediated at least in part by flow‐induced reorganization of ECM fibrils that could promote increased cell‐cell interactions. Surprisingly, culture in 3D ECM gels with superfusion also was required to avoid B cell autoactivation. These responses were likely not due to the effects of direct fluid forces (e.g., shear stress) on the ECM because we used superfusion; however, fluid flow in one channel of the two‐channel microfluidic devices we used in this study in combination with the metabolism of living cells in the parallel channel has been previously shown to be able to generate chemical gradients along the length of the chip that can influence developmental control,^[^ ^46^ ^]^ which could come into play in the LF Chip as well. Together, these findings raise the intriguing possibility that dynamic perfusion of the vasculature of lymph nodes or tertiary lymphoid organs also may actively promote LF formation and growth, and perhaps even contribute to maintenance of a naïve state of these cells in vivo. This hypothesis is consistent with the observation that lymphatic vessel formation accompanies tertiary lymphoid organ formation and that lymph node formation can be inhibited by suppressing lymphatic vessel development.^[^ ^47^, ^48^ ^]^ Physical forces and ECM reorganization underlie many developmental processes^[^ ^49^ ^]^ and the microfluidic LF Chip can be used to explore this mechanochemical mechanism in detail in the future.

Interestingly, we also discovered that high‐density co‐cultures of B and T cells in ECM gels cultured induce AID expression in LF chips, and that larger follicles displayed a higher expression of AID. AID expression was not detected in the donor PBMCs prior to culture and the levels after 2D culture at conventional cell density without ECM or perfusion were not significantly different from those observed in secondary antibody‐only controls. AID was also detected in static 3D culture, but at lower levels than in the LF Chip, which is consistent with minimal follicle formation. Taken together, these data suggest that the high cell density, contact with ECM and/or 3D culture is sufficient to induce low levels of AID expression, however, addition of superfusion as in the LF Chip leads to further upregulation of AID in association with LF formation. AID expression by these cells suggests that they might be in a transitory stage between circulating naïve B cells and activated germinal center cells, which have been recently described as pro‐germinal center cells that are IgD^+^ AID^+^ CD27^−^.^[^ ^50^ ^]^ We also confirmed that there is a link between CXCL13 induction and follicle formation, which has been previously observed during vaccine responses in humans.^[^ ^16^, ^17^ ^]^ In addition, CXCL13 and CXCR5 are highly represented in the genetic signatures of tertiary lymphoid organs as they are in our LF Chips, whereas CXCL13 could not be detected in 2D cultures of naïve lymphocytes. CXCL13 was also expressed in static 3D culture and its levels were, in fact, higher than measured in the LF Chips again suggesting cell density, ECM, and/or 3D culture are critical triggers for acquisition of these features characteristic of lymphocytes within follicles. Yet static 3D cultures autoactivate with high levels of non‐specific IgG production and do not self‐assemble into follicles suggesting that perfusion allows ectopic LF formation in vitro without autoactivation due to culturing cells at tissue like density. While we focused on the development of a human in vitro model of ectopic LF formation and its use for assessing vaccine and adjuvant candidates, it should be possible to define key parameters that govern LF assembly including other relevant cell types (e.g., endothelial cells, stromal cells) in the future using this new experimental platform. The high cell density increases cell‐cell contact formation and leads to tissue‐like polarization, and the 3D culture also enables cell‐ECM contact formation, which could reprogram circulating cells. The commercial ECM gel (Matrigel) also may contain other factors which could contribute to induce AID as well. Understanding the precise molecular basis of these contributions will require additional studies in the future.

Using the LF Chip, we found that challenge with 3 different types of immunological stimuli—IL4 combined with anti‐CD40 Ab, SAC, or the clinically relevant Fluzone vaccine—all promote spontaneous LF development, plasma cell formation, and secretion of antigen‐specific IgG. In contrast, current methods for plasma cell culture rely on extensive cytokine stimulation of memory B cells, stromal cell co‐culture, and multiple rounds of manual handling.^[^ ^36^, ^51^, ^52^, ^53^ ^]^ Treatment of human LF chips with IL‐4 and anti‐CD40 Ab led to robust expansion of plasma cells that were preferentially localized in LFs. It is important to note that other in vitro 3D models of the human lymph node have been explored in the past, such as the MIMIC system that mixes microbeads bound to T or B cells,^[^ ^38^ ^]^ and the HuALN lymph node system that uses a continuously perfused mesh as a tissue‐like support.^[^ ^39^ ^]^ However, neither of these models produce detectable LF formation or robust plasma cell formation. In recently published tonsil organoids,^[^ ^40^ ^]^ plasmablast formation can be observed in response to vaccination, but its formation and the physical and biochemical cues that control it cannot be studied in situ.

Testing new vaccines for emergent influenza virus strains is a huge burden on the healthcare system, as the vaccines must be repeatedly optimized because recombination between influenza virus strains is very common and influenza surface antigens can rapidly mutate. Influenza vaccines, which are currently, tested in ferrets, commonly only result in 30–60% protection in humans.^[^ ^54^ ^]^ Accurate in vitro assessment of vaccines for new strains of influenza using primary human cells that generate plasma cells and undergo Ab class switching would therefore represent a major advance, in addition to significantly reducing the need to rely on animal models. In our studies, we were able to demonstrate successful vaccination in human LF Chips when inoculated with split H5N1 antigen, as measured by increased LF formation, plasma cell differentiation, and anti‐HA IgG production, and that we can detect augmentation of this response by addition of a low cost adjuvant (SWE) in vitro. In contrast, influenza vaccination alone or with SWE only induced modest anti‐HA Ab responses in 2D cultures containing the same cell types, which do not support formation of 3D structures from circulating immune cells. These data clearly show that the formation of 3D LFs within the microfluidic Organ Chip is a significant and novel advance over existing culture and animal models as it recapitulates seasonal vaccine‐induced LF formation and production of antigen‐specific IgG Ab in human‐derived immune cells in vitro. Importantly, when we tested the utility of the human LF chip by vaccinating chips made from 8 independent donors with Fluzone, we detected donor‐specific responses with formation of plasma cells in the high‐responding donors who also produce cytokine biomarkers similar to those seen in vivo. Not surprisingly, we see significant variability between donors in several of the assays (e.g., follicle formation, production of antigen‐specific antibodies). However, as we are not providing any exogenous growth factors with the exception of Matrigel, the number of antigen‐specific precursors could be markedly different between donors and could further change over the course of culture. It is not well understood how immune cells respond to Matrigel and in future studies, additional cytokines and tissue‐specific ECM representing the LF milieu may be added to further increase vaccination‐induced responses in the human LF chip.

Thus, the LF Chip may provide a valuable preclinical tool that can overcome the limitations of humanized mice and even non‐human primates to inform clinical vaccine trials design. In support of this possibility, IFN‐*γ* was upregulated in the LF chip on vaccination as it is in humans, and this has been shown to be a key biomarker of vaccine efficacy.^[^ ^55^ ^]^ As we used bulk B and T cells in the human LF Chips that were vaccinated, it is possible that the high responder cells had a preexisting memory for the Fluzone antigens. However, we chose this strategy as it replicates the administration of seasonal influenza vaccines in human patients who have had varying degrees of past exposure to the vaccine and closely related cross‐reactive viral strains. Finally, the high sensitivity digital anti‐HA ELISA described here can complement traditional methods of quantifying anti‐influenza antibodies, which can predict protection from influenza in seropositive donors, but are severely limited in sensitivity.

In summary, this is the first reported in vitro model that supports formation of human LFs that recapitulate many features of germinal centers found in secondary and tertiary lymphoid organs in vivo using primary human cells obtained non‐invasively from peripheral blood. In addition, we demonstrated that human LF Chips containing B and T cells along with autologous antigen‐presenting DCs can be used to test human immunization responses to vaccines and adjuvants in vitro. In contrast to other culture models, the human LF Chip also can be used to study in vivo‐like ectopic LF formation as well as antigen‐induced immunological responses, and all of this is done using primary human blood cells collected non‐invasively. Key improvements as compared to humanized mice and other existing in vitro systems used to study adaptive immune responses include 3D follicle formation, expression of AID and CXCL13, plasma cell formation, antigen‐specific IgG Ab generation, and production of clinically relevant cytokines in response to multiple antigens, including a commercial influenza vaccine. The human LF Chip enables the longitudinal study of all these responses within a single device during the course of an experiment, and many of the processes can be observed over time in situ using high‐resolution imaging. These microdevices are also patient‐specific and thus, they can recapitulate donor variability in Ab responses to vaccination as seen in our studies with Fluzone. Taken together, these findings suggest that the LF Chip potentially may be useful as a more human‐relevant preclinical tool for assessing the efficacy and safety of vaccines, adjuvants, and immunotherapeutic drugs in a patient‐specific manner.

## 4 Experimental Section

### LF Chip Culture

De‐identified human patient‐derived apheresis collars (by‐product of platelet isolation) were obtained from the Crimson Biomaterials Collection Core Facility under approval obtained from the Institutional Review Board at Harvard University (#22470); informed written consent was not required. PBMCs were isolated by density centrifugation using Lymphoprep (StemCell Technologies, 07801), and magnetic beads were used for negative selection of bulk B cells (StemCell Technologies, 19054) or naïve B cells (17254), T cells (17951) and monocytes (19058). Isolated cells were counted and directly seeded on chip or frozen in Recovery Cell Culture Freezing Medium (Gibco, 12648‐010). Monocytes were differentiated into DCs as previously described.^[^ ^56^ ^]^ Briefly, 1 × 10^6^ monocytes were cultured in complete RPMI (RPMI 1640, Gibco, 72400‐047), 10% Fetal bovine Serum [FBS, Gibco, 10082‐147], 1% Penicillin/Streptomycin (Gibco, 15140122), and 400 ng mL^−1^ GM‐CSF (Mitenyi Biotec, 130‐095‐372) and 250 ng mL^−1^ IL‐4 (130‐094‐117) for 5–6 days with 50% of the medium being replaced with fresh medium and cytokines every 2–3 days.

Organ Chips fabricated as previously described^[^ ^10^ ^]^ or purchased from a commercial vendor (Emulate Inc., Boston, MA) that contain two parallel channels separated by a porous membrane (7 µm pores) were first perfused and incubated with 1% 3‐Aminopropyltrimethoxysilane (Sigma, 281778) in ethanol for 1 h and then incubated in an 80 °C oven overnight. Cells were then suspended (1.5–2 × 10^8^ cells mL^−1^) in complete RPMI containing Matrigel (60%; Corning, 356234) and type I collagen (15%; Corning, 354249) maintained on ice, and the viscous solution was then introduced in the bottom channel of the 2‐channel Organ Chip. After the ECM was allowed to gel for 30 min in a 37 °C cell culture incubator, the top channel of the chip was filled with medium and channel entry and exit ports were plugged with 200 µL tips containing medium. The following day chips were perfused with RPMI medium containing at 60 µL h^−1^ using peristaltic pumps (Cole Parmer) or automated Zoe Organ Chip instruments (purchased from Emulate Inc.) in a 37 °C cell culture incubator following the manufacturer's instructions. Both chip sources and perfusion systems produced similar results.

### 3D Cell Culture

To study immune response in conventional 2D culture and to compare the results to the Human LF chip, the lymphocyte mix was resuspended in medium at 2.25–3×10^6^ cells mL^−1^ and plated at 1 mL well^−1^ in tissue culture treated 24 well plates or 0.5 mL well^−1^ in 48 well plates. Vaccination was performed by adding vaccine with or without adjuvant in an equal volume for a total volume of 2 mL well^−1^ in a 24 well plate or 1 mL well^−1^ in a 48 well plate. To compare results between 2D culture and chip, cytokine and Ab results were normalized for volume and cell number.

### Activation of Human LF Chip

To activate immune responses, chips were treated with immune activators by perfusing medium containing SAC (Sigma, 1:50 000), IL4 (Miltenyi Biotec, 130‐094‐117, 400 µ mL^−1^) and anti‐CD40 Ab (BioXcell, BE0189, 1 µg mL^−1^]), or Fluzone (BEI resources, NR‐19879, 10 µL mL^−1^) through the top channel of the device. Treatment with IL4 and anti‐CD40 was initiated 2 days after seeding and SAC treatment was initiated 4 days after seeding (3 days of perfusion), and fresh medium was added to the inlet perfusion reservoirs as needed. In the Fluzone experiments, medium was recirculated for the first 5 days of treatment (i.e., effluents were added back to the inlet perfusion reservoir), and at day 5 and every 3–4 days thereafter, a 1:1 mix of effluent and fresh medium was used for perfusion. This was done to ensure that the cytokine milieu was maintained and to limit and taper the amount of Fluzone consumed by the experiment.

The following reagents were obtained through BEI Resources, NIAID, NIH: Fluzone® trivalent seasonal Influenza Virus Vaccine, 2009–2010 Formula, NR‐19879 and HA1 Hemagglutinin (HA) Protein with N‐Terminal Histidine Tag from Influenza Virus, A/Brisbane/59/2007 (H1N1), Recombinant from Baculovirus, NR‐13411. Split H5N1 (A/turkey/Turkey/1/2005) pandemic influenza antigen (H5N1) and SWE adjuvant were provided by Vaccine Formulation Institute (VFI) (Plan‐les‐Ouates, Switzerland).^[^ ^57^ ^]^ SWE and H5N1 were resuspended to an isotonic suspension using buffers provided by VFI. LF chips were vaccinated with 0.1 µg mL^−1^ HA units and 10 µL mL^−1^ SWE.

### Immunofluorescence Microscopy

Samples were stained with a fluorophore‐labeled antibodies directed against CD3 (Biolegend, clone UCHT1), AID (Invitrogen, PA5‐20012), or CD138 (Invitrogen, PA5‐47395). To label B and T‐cells for microscopy before seeding chips, lymphocytes were labeled with CellTracker dyes (Invitrogen, 1–2 µM). LF chips were fixed by filling the perfusion channel with 4% paraformaldehyde in phosphate‐buffered saline (PBS, 1 h at room temperature, RT) and plugging the channel entry and exit ports with 200 µL tips containing fixative. To carry out immunostaining, chips were similarly incubated with primary antibodies diluted in PBS containing 1% FBS and Fc Block (1:20 dilution; Miltenyi Biotec, 130‐059‐901) overnight at RT, washed several times with PBS, and then incubated with secondary antibodies diluted in PBS containing 1% FBS overnight at RT, followed by 1‐h incubation with Hoechst (Life Technologies, H3570, 1:1000) and several PBS washes at RT. ECM fibers were imaged using second harmonic imaging after fixation which allows the label‐free imaging of biopolymers using a Leica SP5 confocal microscope. Follicle size and number were quantified using the surfaces function of Imaris (Bitplane) software (Figure S1d, Supporting Information). For analysis of CD138 and AID in single cells and follicles, regions of interest were defined in ImageJ using a cellular counterstain such as Hoechst. Area or MFI of expression were then quantified in these regions. For comparison of AID expression levels between static 3D cultures and LF chips, the MFI of the whole field of view was measured. To estimate the numbers of AID+ cells within individual follicles, we quantified the % of the projected area of each LF expressing AID in perfused Organ Chips cultured for 4 days.

### Quantification of T Cell Polarization

Using ImageJ software, random cells were selected, and the center of mass of each cell was defined. Next, the center of mass of the CD3‐positive fluorescent region was determined for each cell, and then the absolute distance between the center of the cell and the center of the CD3‐stained region was used to define T cell polarization, with greater distances indicating more polarization.

### Flow Cytometry

Cells were harvested from chips by blocking one port of the basal channel with a tip and manually pipetting Cell Recovery Medium (Corning, 354253 200 µL chip^−1^) through the other port to push the ECM and cells out of the channel and harvest them. The harvested ECM was incubated in the Cell Recovery Medium for 1 h at 4 °C. The released single cells were centrifuged at 300 g for 5 min and resuspended in PBS for staining. Cells were first labeled with Live/Dead fixable dyes (1:1000 dilution; Invitrogen, L34963), followed by a 15 min incubation with fluorophore‐labeled antibodies and Fc Block (1:100 dilution) at 4 °C. Cells were washed twice and then fixed with Cytofix (BD Biosciences, 554655) for 15 min at RT. Cells were centrifuged and re‐suspended in PBS and stored at 4 °C prior to cytometric analysis using a using LSRFortessa flow analyzer (BD Biosciences). Results were analyzed using FlowJo V10 software (Flowjo, LLC) using a volume of 100 uL containing between 0.5 to 1 × 10^6^ cells. Antibodies to CD19 (#340437), CD27 (#655429), CD3 (#641406), CD4 (#555346), and CD8 (#347314) were obtained from BD Biosciences; antibodies to IgD (#348210) and CXCR5 (#356919) were acquired from BioLegend.

### Immunoglobulin and Cytokine Quantification

Total immunoglobulin levels were measured using ELISA [Bethyl Biolabs, E80‐104 or Mesoscale discovery, K15203D). Influenza HA‐specific IgG was detected using a modified version of a previously described digital ELISA assay.^[^ ^31^ ^]^ Briefly, HA from Influenza A/59/Brisbane (BEI Resources, NR‐13411) was conjugated to carboxyl‐modified paramagnetic beads. Tonsils cultures (2.25–4 × 10^6^ cells well^−1^ of a 24 well plate) were immunized with 10 uL mL^−1^ of Fluzone^[^ ^32^ ^]^ were used as positive controls. Discarded de‐identified tonsils were obtained from Massachusetts General Hospital (MGH) under an Institutional Review Board approved protocol (#2015P001859); informed consent was not required. For detection of anti‐HA antibodies, tonsil culture supernatants or chip effluents were incubated with the beads, followed by a biotinylated anti‐human Ab [Life Technologies, A18821] and streptavidin‐*β*‐galactosidase and analyzed using Simoa HD‐1 Analyzer (Quanterix). The sandwich digital ELISA was also used with antibodies from Biolegend directed against to IFN‐*γ* (#507502), IL‐7 (#501302), or IL‐10 (#50680) and from R&D Systems against IL‐2 (#MAB602), GM‐CSF (#MAB615), or CXCL13 (#DY801). Secondary antibodies used in these studies against IL 10 (#501501) and IL‐7 (#506601) were from Biolegend, and against IFN‐*γ* (#MAB285), IL‐2 (#MAB202), GM‐CSF (#BAF215), CXCL13 (#DY801) were from R&D Systems. Protein standards for recombinant IFN‐*γ* (#285‐IF‐100), IL‐2 (#202‐IL‐010), IL‐7 (#207‐IL‐005), IL‐10 (#217‐IL‐005), GM‐CSF (#215‐GM‐010), CXCL13 (#DY801) were all from R&D Systems.

Briefly, immune complexes were formed on beads in a 3‐step format by 1) capturing target with Ab‐conjugated beads, 2) binding with biotinylated detection Ab, and 3) labeling with streptavidin‐*β*‐galactosidase. In depth descriptions of Simoa assay development and performance have been previously reported.^[^ ^31^ ^]^ Participant recruitment and sample collection followed an Institutional Review Board (IRB, Tufts University)‐approved protocol (Study number 1410019). Consent was not required for use of blood samples in the current study. They were excess de‐identified samples. Secondary use was not prohibited.

Results obtained from immunoassays comparing 2D supernatants and LF Chip cultures were normalized for medium volumes and starting cell numbers. Static 3D cultures were maintained in a volume of 0.25ml as compared to human LF chip cultures which were perfused with 2 mL (Figure 1f). For comparison of secreted factors between static 3D and LF Chip cultures, the values obtained for static 3D cultures were divided by 8 to normalize for medium volume.

2D cultures were initiated in 2 mL of medium similar to LF chips. The medium was recirculated for the first 5 days of Fluzone immunization in human LF chips (i.e., effluents were added back to the inlet perfusion reservoir). On day 5, 0.5 mL of the medium was collected for biomarker analyses and 1.5 mL of fresh medium was added to the human LF chip (total chip perfusion volume = 3 mL) and recirculated by transferring medium from the outlet to the inlet reservoir as needed until day 9. To maintain similar levels of analyte dilution, 50% of the medium from 2D cultures (1 mL) was also replaced with fresh medium (total 2D culture volume = 2 mL). For the day 9 antibody analyses, media from chip outlet and inlet reservoirs were pooled (total volume = 3 mL) and samples were collected for analyses (**Table** **1**).

**Table 1:** Culture volume and replacements in 2D cultures and LF chips

Culture type	Starting number of cells	Starting volume	Day 5 medium removed for cytokine analysis	Day 5 fresh medium added	Day 9 collection volume for antibody analysis	Dilution factor of LF chip vs. 2D
2D	2.25 × 10^6^	2 mL	1 mL	1 mL	2 mL	
LF Chip	2.25 × 10^6^	2 mL	0.5 mL	1.5 mL	3 mL	1.5x

For analysis of anti‐HA antibodies in Figure 4a, media samples diluted 4 times were used to measure antibodies in the SiMoa assay. A calibration curve between AEB signal and sample concentration was generated by diluting a control sample and plotting the observed AEB signal versus sample concentration in Graphpad Prism. There was a linear relationship between AEB and sample concentration [AEB signal = 0.2124(sample concentration)+ 0.01003]. LOD was defined as 3× standard deviation (AEB of medium alone) + average (AEB of medium alone).

Using the linear equation defined above, AEB values for chip were normalized for the different media volume per the following equation if the observed AEB value was above the LOD values that were detectable (i.e., above the LOD) were normalized as shown below:

(1) $$\begin{array}{ccc} & & \mathrm{Normalized}\;\mathrm{AEB}\;\mathrm{value}\;\mathrm{for}\;\mathrm{LF}\;\mathrm{chips}\\ & & \;=[\left(\mathrm{observed}\;\mathrm{AEB}\;\mathrm{value}-0.01003\right)*1.5*4+0.01003 \end{array}$$

(2) $$\begin{array}{ccc} & & \mathrm{Normalized}\;\mathrm{AEB}\;\mathrm{value}\;\mathrm{for}\;2D\;\mathrm{cultures}\\ & & \;=[\left(\mathrm{observed}\;\mathrm{AEB}\;\mathrm{value}-0.01003\right)*0.01003 \end{array}$$

### Statistical Analyses

Tests for linear correlations and statistical significance were performed using Prism (GraphPad Software). For comparison of 2 datasets, Student's *t*‐test was used; if SD was not equal, Welch's correction was used. For comparison of 3 or more datasets where distribution of data could be assumed to be normal one‐way ANOVA followed by a Fisher's LSD test was used except in Figure S4c, Supporting Information, where Sidak's multiple testing correction was applied. If SD was not equal for 3 or more independent groups, Brown‐Forsythe one way ANOVA with an unpaired t‐test with Welch's correction was used. For data that did not have a normal distribution, Kruskal–Wallis test for unpaired data and Friedman test for paired data were performed followed by an uncorrected Dunn's test. For grouped data where two factors were considered, for example, single versus clustered cells from two different activation states, two‐way ANOVA was performed followed by Fisher's LSD test. In all figures, * indicates *p* < 0.05 in all figures. The number of donors, chips/donor, and replicate experiments are described in the figure legends.

## Conflict of Interest

D.E.I. is a founder, board member, scientific advisory board chair, and equity holder in Emulate, Inc.; G.G. and D.E.I. are co‐inventors on relevant patent applications. D.R.W. is a founder and has a financial interest in Quanterix Corporation and serves on its Board of Directors.

## Author Contributions

G.G. designed in vitro experiments with the help of G.M. G.G, G.M., and P.P performed in vitro experiments and analyzed the data, working with D.E.I, who also supervised all work. Y.Z, M.S.K, A.M, A.P, A.J, T.C.F, D.C, Y.Z., and J.L. assisted in performing experiments and analyzing the data. B.B, L.X, T.G, R.L., and L.M. designed and conducted all high sensitivity digital ELISAs under the guidance of D.R.W. G.G, G.M, Y.Z., and D.E.I. wrote the manuscript.

## Supporting information

Supporting Information

Supporting Figure

Supporting Table

## Data Availability

The data that support the findings of this study are available from the corresponding author upon reasonable request.
