# Supplementary material for: Ectopic Lymphoid Follicle Formation and Human Seasonal Influenza Vaccination Responses Recapitulated in an Organ‐on‐a‐Chip
Source: Adv Sci (Weinh). 2022 Mar 14;9(14):2103241. doi: 10.1002/advs.202103241 (PMC9109055; doi:10.1002/advs.202103241)
Supplement: Supplementary file 1 — Supporting Information [file ADVS-9-2103241-s001.pdf]

Supporting Information

for *Adv. Sci.,* DOI: 10.1002/advs.202103241

Ectopic lymphoid Follicle Formation and Human Seasonal Influenza Vaccination Responses Recapitulated in an Organ-on-a-chip

G. Goyal[1], P. Prabhala[1], G. Mahajan[1], B. Bausk[1,2], T. Gilboa[1,2], L. Xie[1,2], Y. Zhai[1], R. Lazarovits[1], A. Mansour[1], Min Sun Kim[1], A. Patil[1], D. Curran[1], J. M. Long[1], S. Sharma[1], A. Junaid[1], L. Cohen[1,2], T.C. Ferrante[1], O. Levy[1], R. Prantil-Baun[1], D.R. Walt[1,2] and D.E. Ingber[1,3,4]*

SUPPLEMENTARY TABLE & FIGURE LEGENDS

Supplementary Table 1. Clinical Samples used in this Study.

Supplementary Table 2. Summary of Culture Systems and Results. Various characteristics and phenotypes described were assessed in different culture systems without (-) or with the listed stimulants. Results were scored qualitatively and refer to either a low level of phenotypic response in some (+/-) or all (+) donors, or a high level of response in all donors (++).

Supplementary Figure 1. **a)** 3D drawing of Organ Chip with the channel containing the 3D ECM gel colored in orange; the parallel flow channel is not colored. **b)** Immunofluorescence micrographs showing CellTracker labeled human B (magenta) and T lymphocytes (green) cultured within an ECM gel in the LF Chip at day 0 and 4 (3 days after perfusion was initiated); bar, 50 μm). **c)** Representative surfaces seen on the LN chip after 4 days of culture visualized as surfaces with Imaris software. 1 field from a representative donor shown (similar results were obtained in 6 donors). **d)** Change in follicle number over time in 2 donors. Each dot represents one field of view (n>6). Donors are distinguished by color of the dot. *, $p < 0.05$ using a one way ANOVA followed by the Fisher's LSD test. **e)** Confocal immunofluorescence micrographs of T cells in 2D culture (2D) versus perfused LF chip (chip) stained for CD3 (left), and quantification of T cell polarization carried out in 2D culture, static organ chips or the perfused LF chip (right) as described in Methods. Each dot is a cell and >63 cells were analyzed in all conditions. Results from one donor are shown, and similar results were obtained in LF Chips created with cells from three different donors. Error bars indicate SD; *, $p < 0.05$ using a Browne-Forsythe one way ANOVA followed by unpaired t-test with Welch's correction for unequal variances. **f)** CXCL13 levels in static 3D cultures of 3 donors. Donors are distinguished by color of the dot.

Supplementary Figure 2. Schematic of culture systems used in this study in Figures 1-3 and Supplementary Figures S1-S4

Supplementary Figure 3. a) Purity of naïve B cells sorted from apheresis collars. 1 representative donor shown out of >6 donors tested. **b)** IgM levels as detected by ELISA (limit of detection = 49 pg/ml) by unstimulated static cultures (Static) and perfused chips (Flow) after 7 days of culture. Mean IgM levels from 2 chips/condition from 2 donors (n=4) shown here. Error bars indicate SD; *, $p < 0.05$ using an unpaired Student's t-test with Welch's correction.

Supplementary Figure 4. Optimization of AID staining for confocal microscopy. **a)** AID staining was tested on two donors after 4 days of static culture. **b)** MFI of AID as detected by flow cytometry. Each data point represents 1 donor. One-way ANOVA was performed with post-hoc testing with Sidak correction for multiple testing; *, $p < 0.05$. **c)** The number of AID^+ B cells in individual follicles was estimated by measuring the % projected area of each LF that contained AID expressing cells plotted as a function of LF size (Follicle area) in perfused Organ Chips cultured for 4 days. Each data point represents 1 follicle and follicles from 3 donors (indicated by different colored symbols) were pooled for this analysis. Non parametric Spearman correlation results are shown. **d**) AID MFI from n>4 fields of view in a representative donor in static (Static) and perfused chips (Flow) after 4 days of culture (similar results obtained in 3 donors). Error bars indicate SD. Unpaired Student's t-test, *, $p < 0.05$.

Supplementary Figure 5. Flow cytometric analyses of the human LF chip. a) Dot plots of chips stained with a viability dye and fluorophore labeled antibodies against CD19, CD3, CD4, CD8, CXCR5, IgM, IgG and PD-1. **b)** An equal fraction of the digested chips or 2D culture wells from 4 donors (indicated by different colored symbols) was analyzed by flow cytometry in the presence of counting beads. Number of live cells (Total Cells), total CD19+ B cells (B cells), IgM+ naïve B cells (IgM), IgG+ B cells (IgG), CXCR5+ B cells (CXCR5B), CD3+ T cells (T cells), and PD1+ CXCR5+ T follicular helper cells (Tfh) are shown. *, $p < 0.05$ using an unpaired Student's t-test with Welch's correction.

Supplementary Figure 6. Influenza vaccination on LF chip and measurement of anti-influenza Ab. a) Schematic of culture systems used in Figures 4, 5 and Supplementary Figs S6, S7 for testing influenza vaccines. **b)** Average Enzymes per Bead (AEB) obtained in a digital ELISA at different dilutions of supernatants from tonsil cells from 2 different donors vaccinated with trivalent split virion Fluzone or medium control. **c**) 2-6 replicates of 2D wells of LF Chips were set up for 4 donors with split H5N1 with or without SWE. Percentage of culture wells or chips from each donor that displayed AEB signal above LOD for anti-HA antibodies shown. Friedman's test was used to perform a paired one-way ANOVA followed by an uncorrected Dunn's test; *, $p < 0.05$.

Supplementary Figure 7. Effect of SWE on sustaining LFs. Follicle number (#, left) from 2 independent fields of view each from 2 LF chips (n=4) from a donor vaccinated with split virion H5N1 alone or with 10 μl/ml SWE. Follicle Volume (Vol., right) was also measured; each dot represents one follicles; error bars indicate SD; *, $p < 0.05$ using an unpaired Student's t-test with Welch's correction.
