# Supplementary material for: Ectopic Lymphoid Follicle Formation and Human Seasonal Influenza Vaccination Responses Recapitulated in an Organ‐on‐a‐Chip
Source: Adv Sci (Weinh). 2022 Mar 14;9(14):2103241. doi: 10.1002/advs.202103241 (PMC9109055; doi:10.1002/advs.202103241)
Supplement: Supplementary file 2 — Supporting Figure [file ADVS-9-2103241-s002.pptx]

## Slide 1
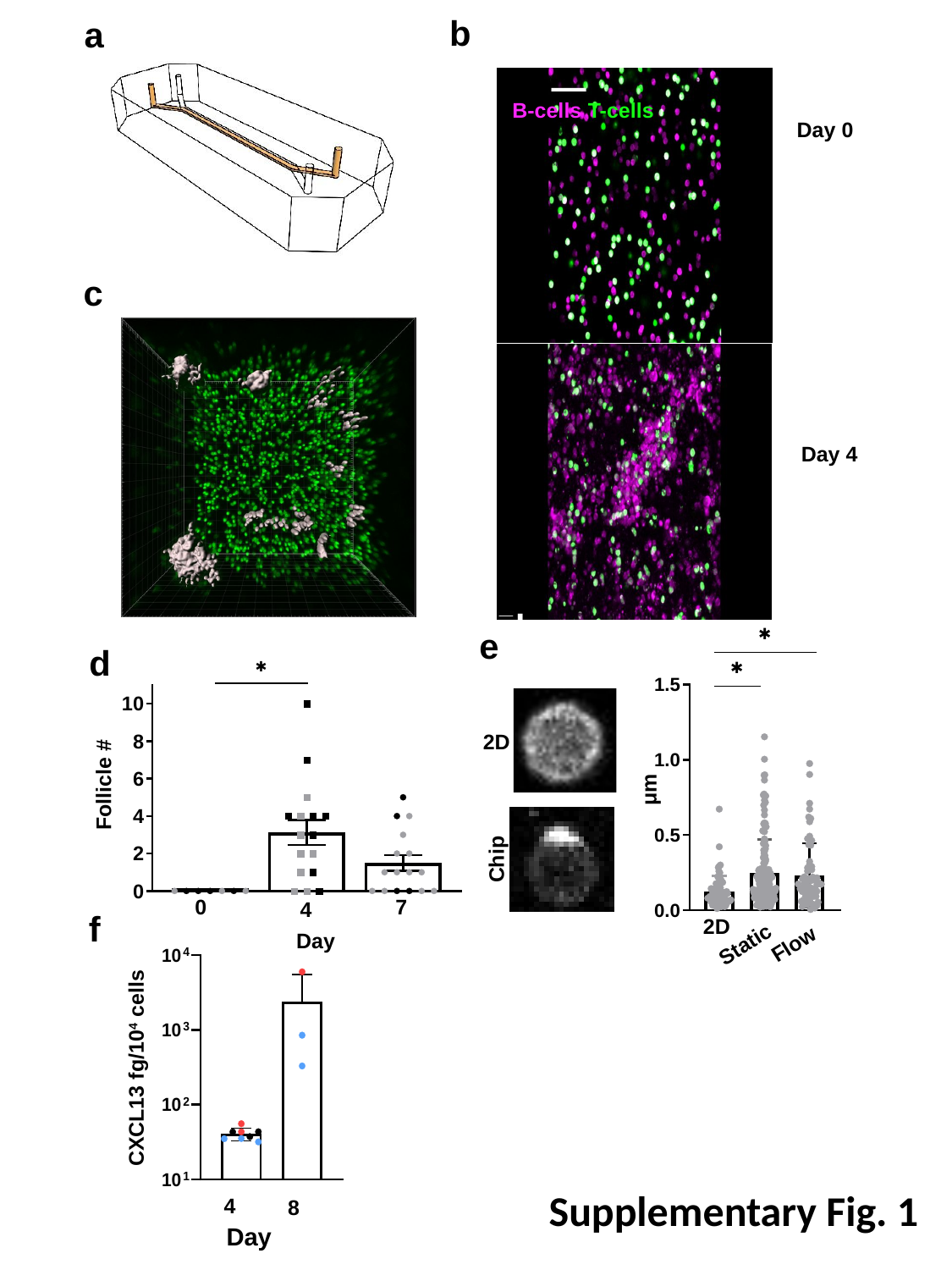

b
a
B-cells T-cells
Day 0
Day 4
c
d
Follicle #
7
0
4
Day
e
d
2D
Chip
μm
2D
Static
Flow
f
CXCL13 fg/104 cells
4
8
Day
Supplementary Fig. 1

## Slide 2
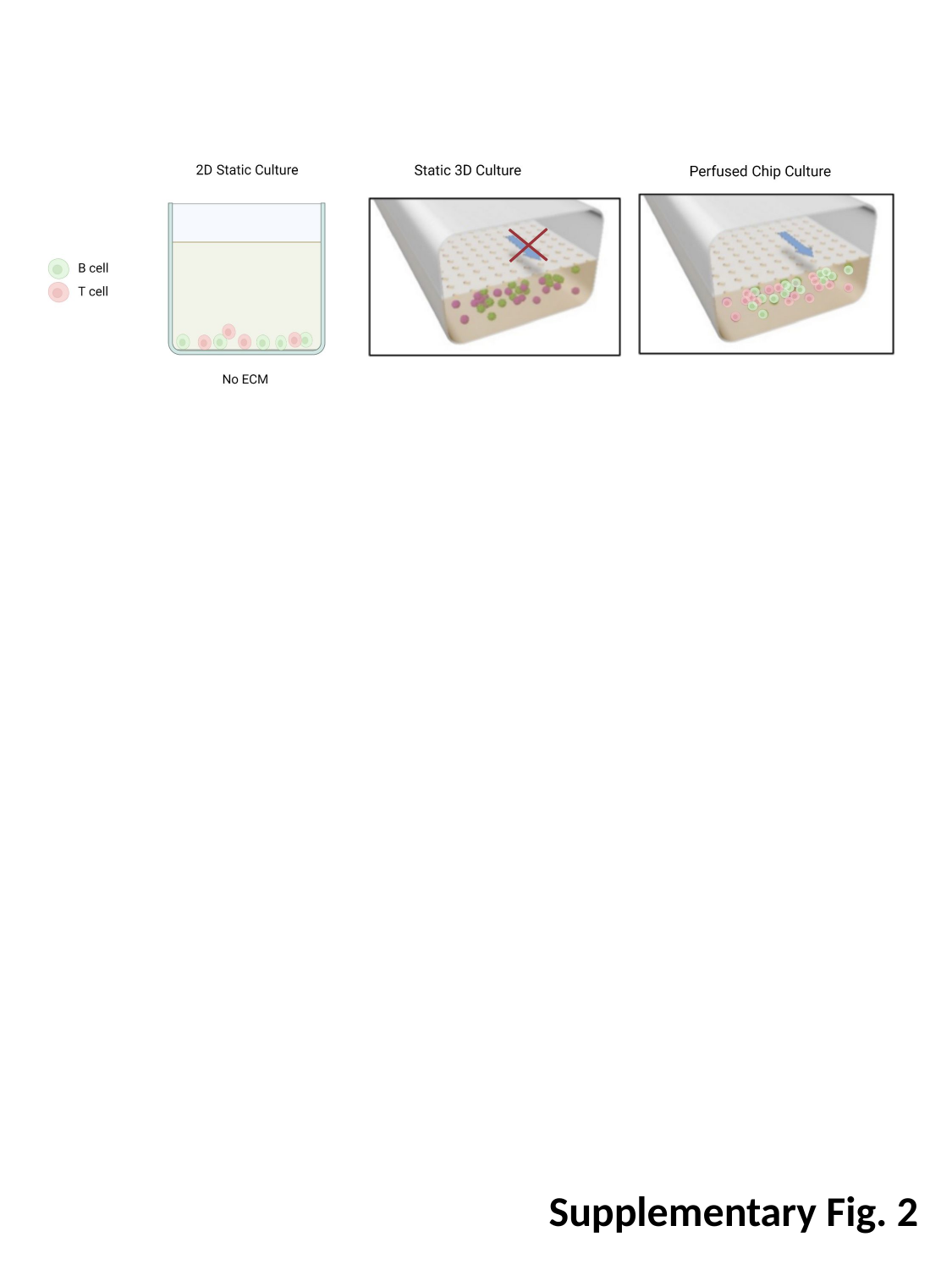

Supplementary Fig. 2

## Slide 3
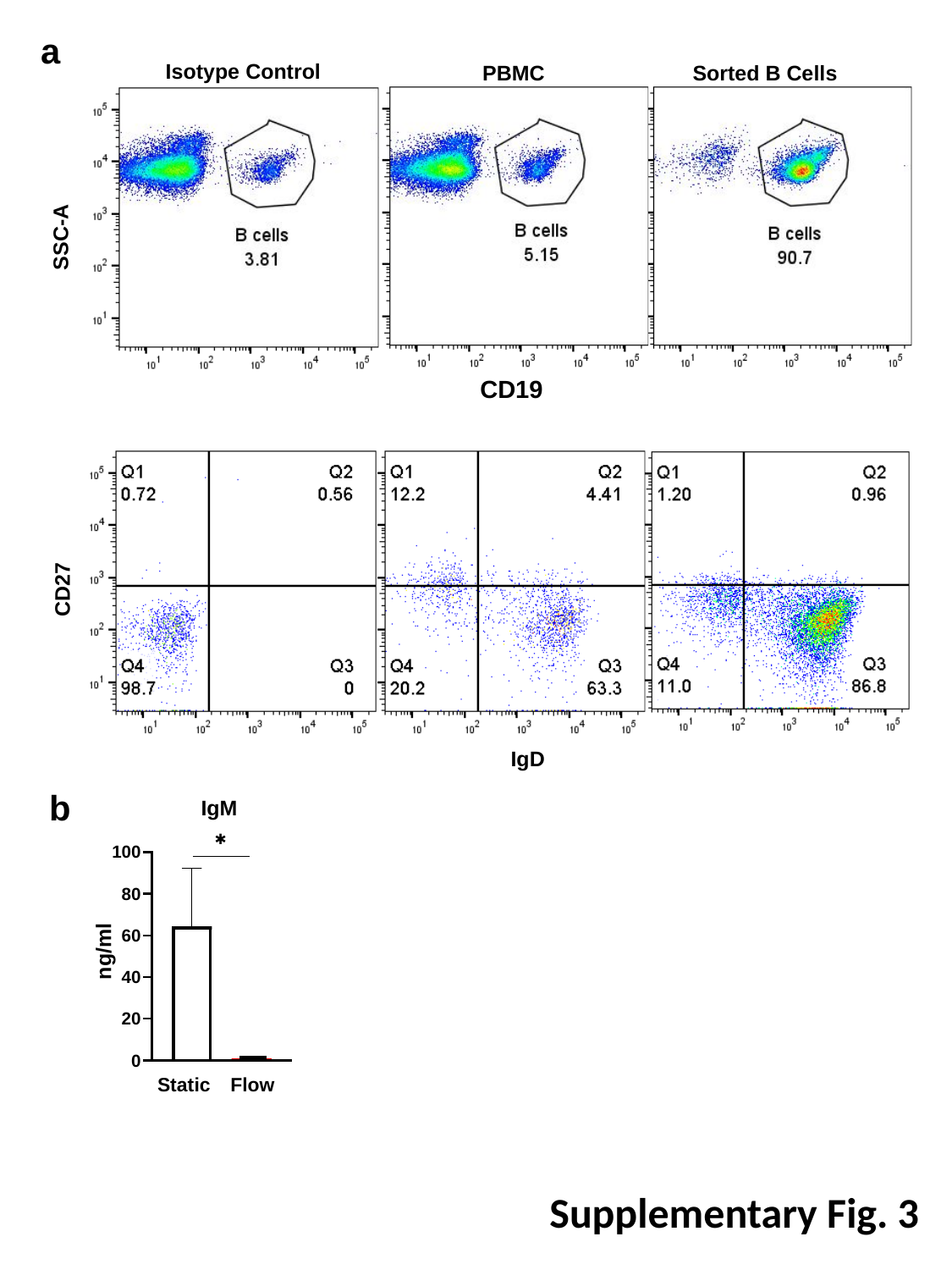

a
Isotype Control
PBMC
Sorted B Cells
SSC-A
CD19
CD27
IgD
b
IgM
ng/ml
Static
Flow
Supplementary Fig. 3

## Slide 4
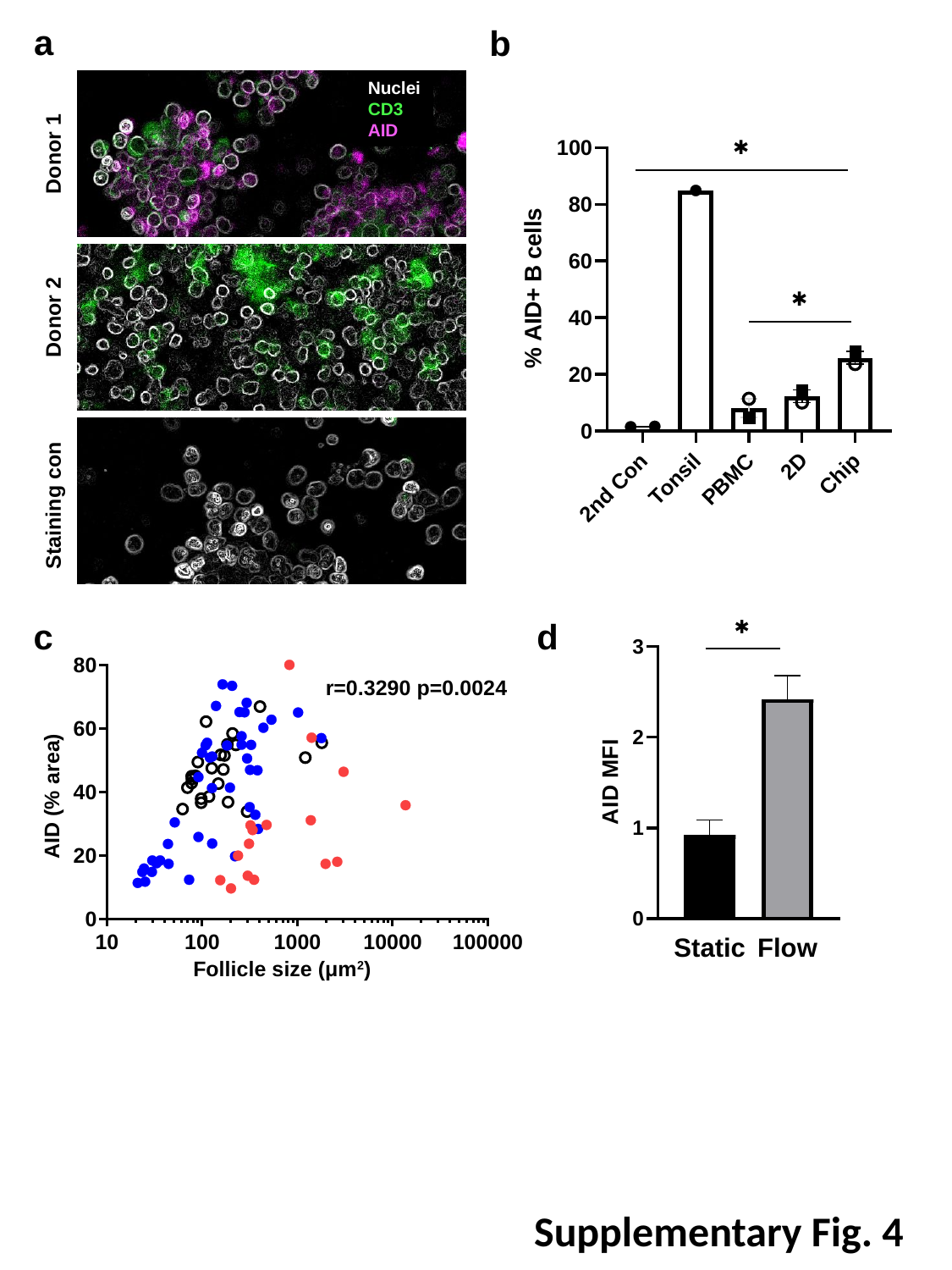

a
b
Nuclei
CD3
AID
Donor 1
Donor 2
Staining con
c
d
r=0.3290 p=0.0024
AID (% area)
Follicle size (μm2)
Supplementary Fig. 4

## Slide 5
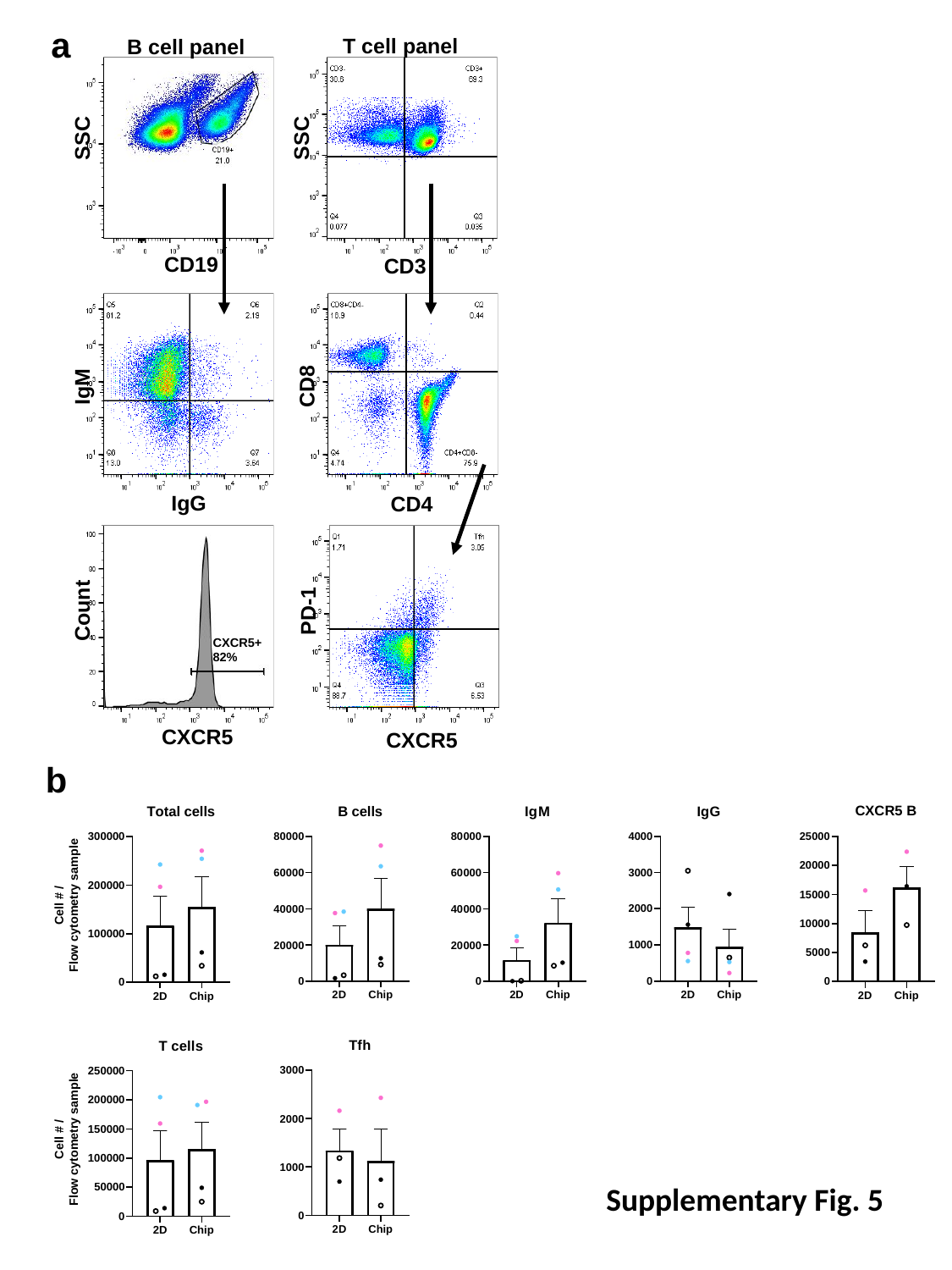

a
T cell panel
B cell panel
SSC
SSC
CD19
CD3
IgM
CD8
IgG
CD4
PD-1
Count
CXCR5
CXCR5
CXCR5+
82%
b
Supplementary Fig. 5

## Slide 6
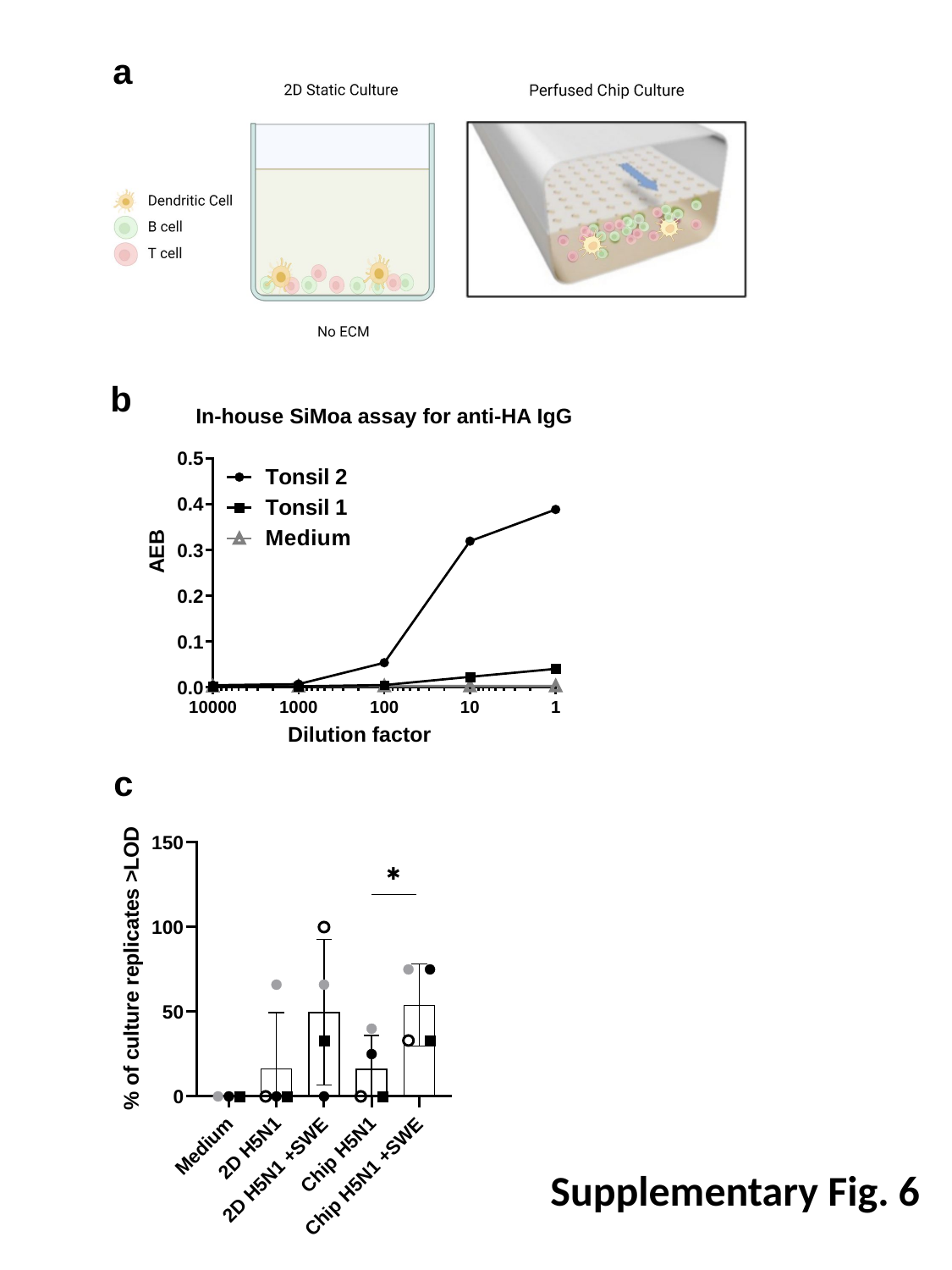

a
b
In-house SiMoa assay for anti-HA IgG
AEB
Dilution factor
c
Supplementary Fig. 6

## Slide 7
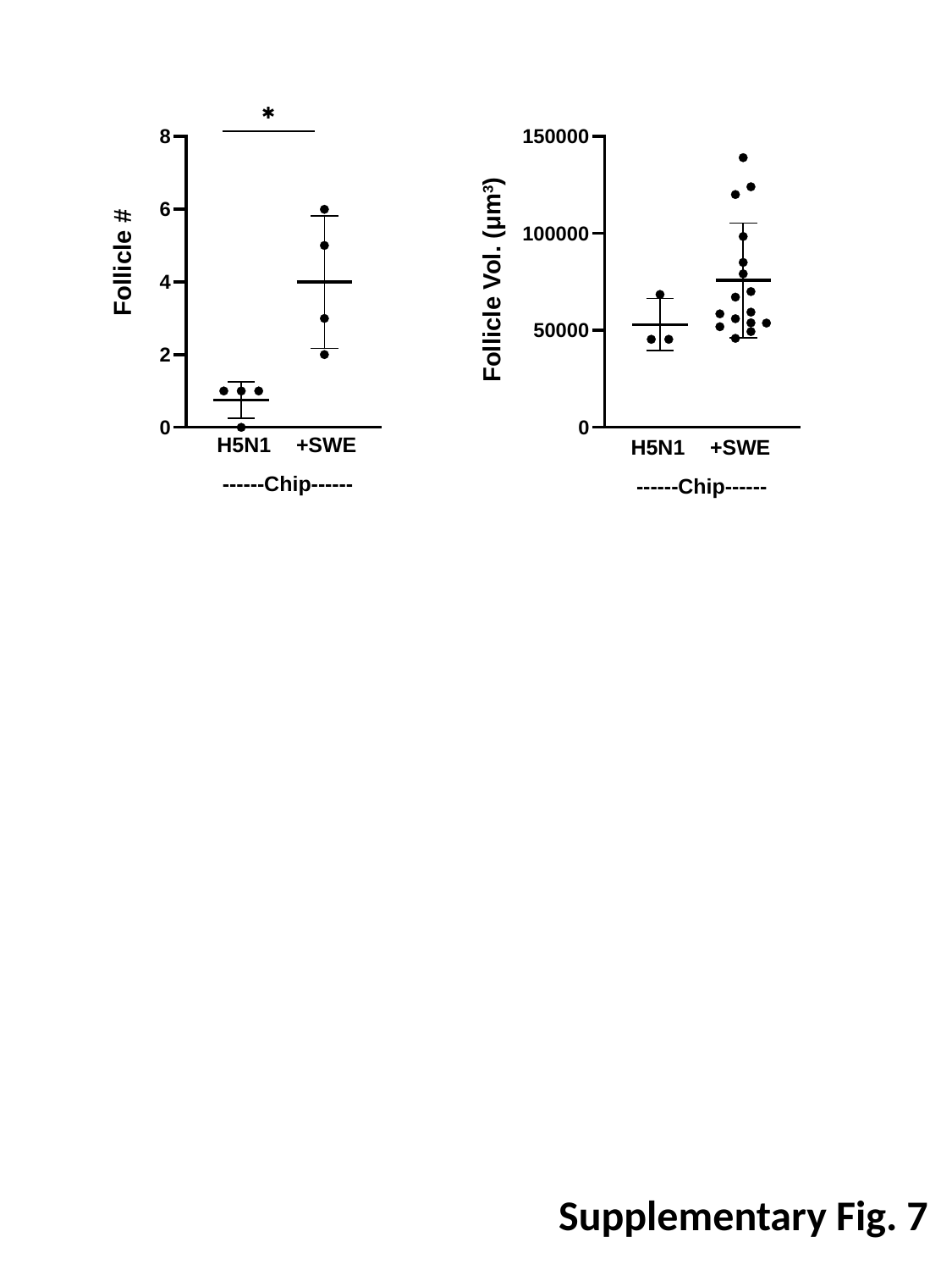

Follicle Vol. (μm3)
Follicle #
H5N1
+SWE
H5N1
+SWE
------Chip------
------Chip------
Supplementary Fig. 7
