# Supplementary material for: Ectopic Lymphoid Follicle Formation and Human Seasonal Influenza Vaccination Responses Recapitulated in an Organ‐on‐a‐Chip
Source: Adv Sci (Weinh). 2022 Mar 14;9(14):2103241. doi: 10.1002/advs.202103241 (PMC9109055; doi:10.1002/advs.202103241)
Supplement: Supplementary file 3 — Supporting Table [file ADVS-9-2103241-s003.pptx]

## Slide 1
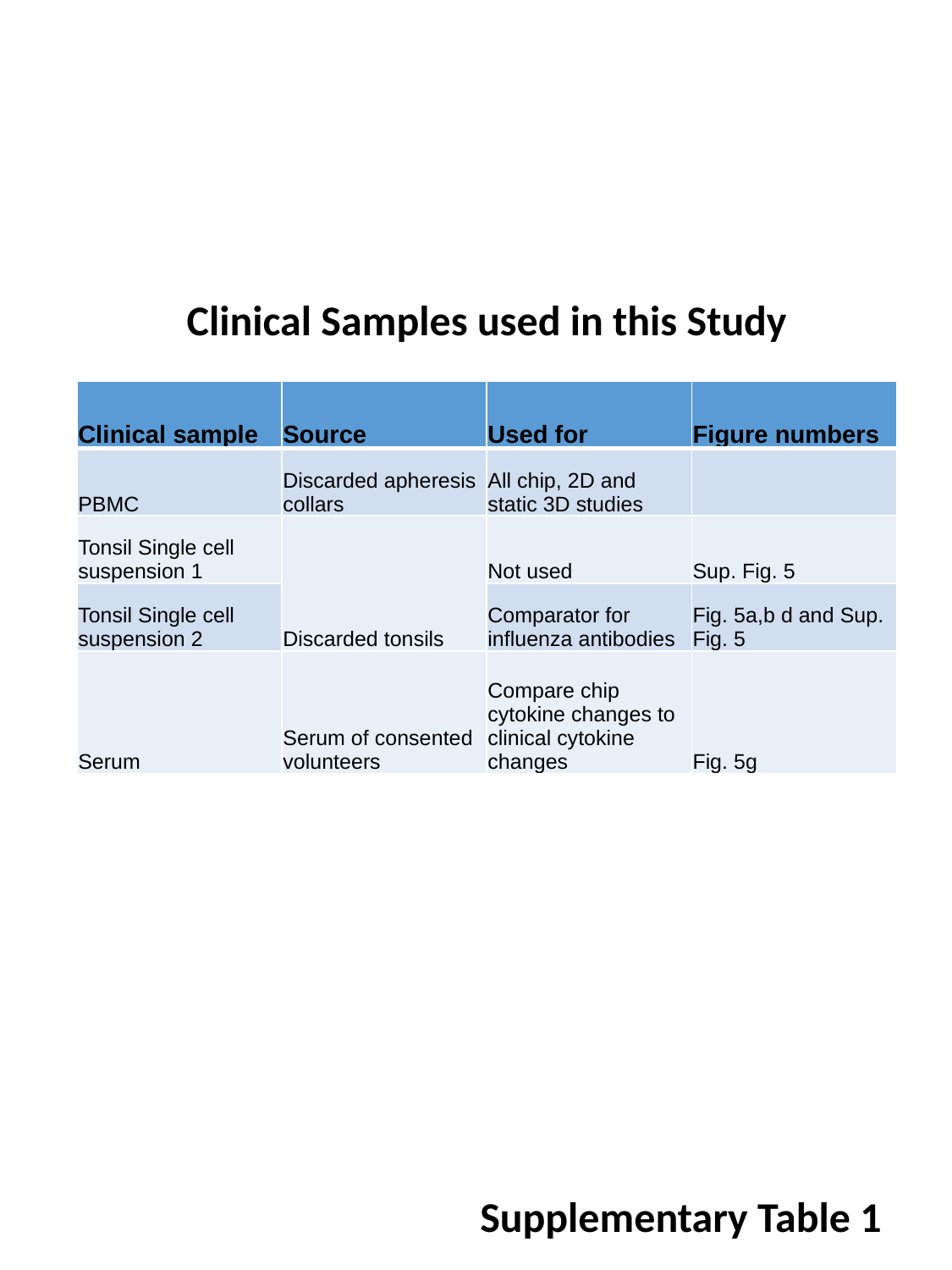

Clinical Samples used in this Study
| Clinical sample | Source | Used for | Figure numbers |
| --- | --- | --- | --- |
| PBMC | Discarded apheresis collars | All chip, 2D and static 3D studies | |
| Tonsil Single cell suspension 1 | Discarded tonsils | Not used | Sup. Fig. 5 |
| Tonsil Single cell suspension 2 | | Comparator for influenza antibodies | Fig. 5a,b d and Sup. Fig. 5 |
| Serum | Serum of consented volunteers | Compare chip cytokine changes to clinical cytokine changes | Fig. 5g |
Supplementary Table 1

## Slide 2
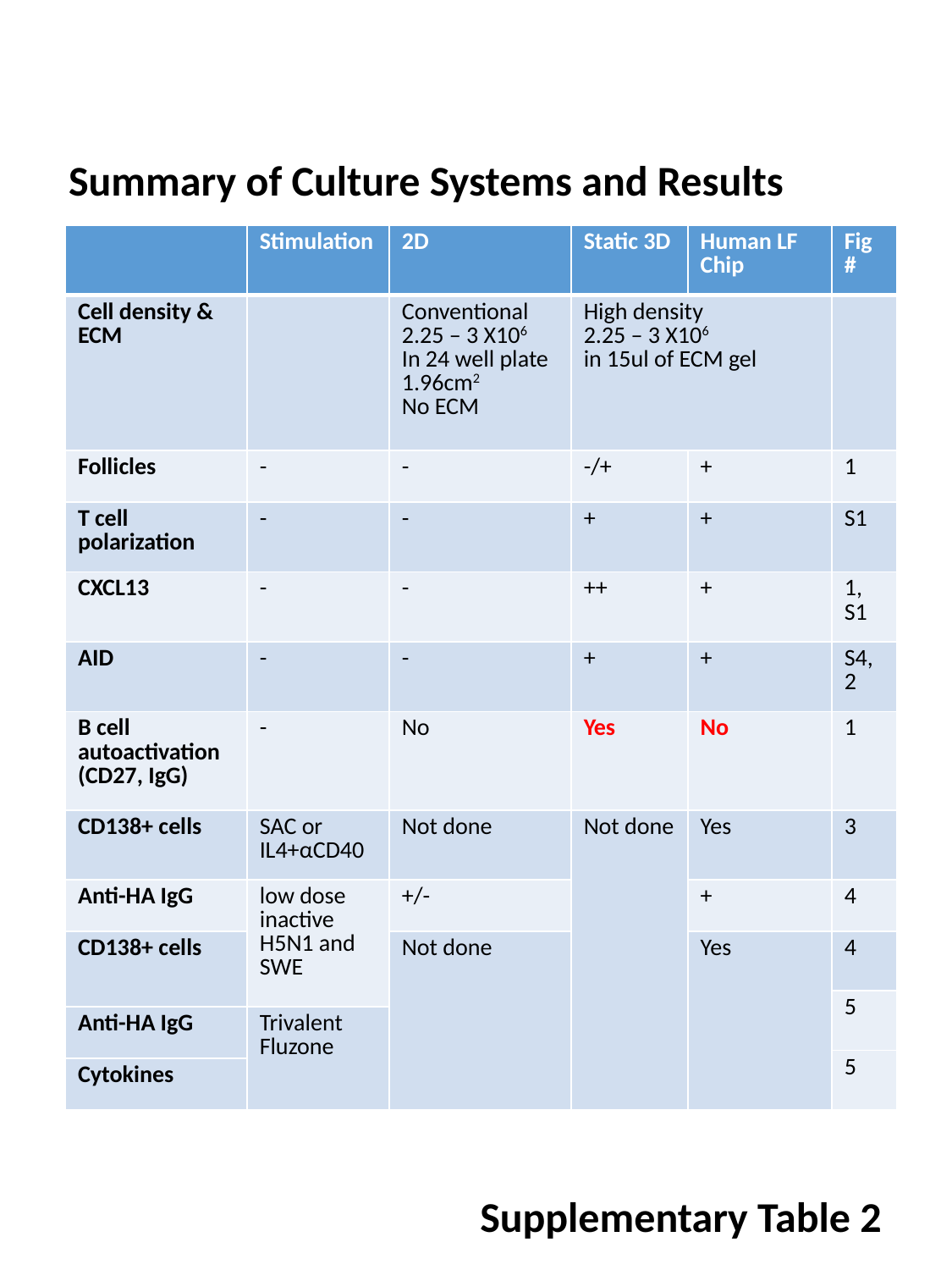

Summary of Culture Systems and Results
| | Stimulation | 2D | Static 3D | Human LF Chip | Fig # |
| --- | --- | --- | --- | --- | --- |
| Cell density & ECM | | Conventional 2.25 – 3 X106 In 24 well plate 1.96cm2 No ECM | High density 2.25 – 3 X106 in 15ul of ECM gel | | |
| Follicles | - | - | -/+ | + | 1 |
| T cell polarization | - | - | + | + | S1 |
| CXCL13 | - | - | ++ | + | 1, S1 |
| AID | - | - | + | + | S4, 2 |
| B cell autoactivation (CD27, IgG) | - | No | Yes | No | 1 |
| CD138+ cells | SAC or IL4+αCD40 | Not done | Not done | Yes | 3 |
| Anti-HA IgG | low dose inactive H5N1 and SWE | +/- | | + | 4 |
| CD138+ cells | | Not done | | Yes | 4 |
| | | | | | 5 |
| Anti-HA IgG | Trivalent Fluzone | | | | |
| | | | | | 5 |
| Cytokines | | | | | |
Supplementary Table 2
